# Civil Disobedience in Democratic Education

**DOI:** 10.1007/s12124-024-09888-y

**Published:** 2025-01-15

**Authors:** Eugene Matusov

**Affiliations:** University of Delaware, Newark, DE USA

**Keywords:** Authorial agency, Democracy, Values, Power, Dissent, Personal responsibility, Democratic governance

## Abstract

The study’s goal was to examine the tension between democratic school governance, requiring its participants to obey school rules, even though they might disagree with those rules, and personal responsibility, requiring the participants to act morally, in accordance with their conscience and their sense of what is right and makes sense for them, regardless of the democratic nature of the imposed school rules. This examination was based on three sources: 1) three Open Symposia with American and Russian democratic educationalists, 2) my review of the existing literature on civil disobedience and democratic education, and 3) my empirical study based on the interviews of participants of an American private democratic school, known as The Circle School, regarding their instances of civil disobedience. The three Open Symposia allowed me to develop a working definition of civil disobedience as a particular principled disobedience. One important finding arising from these Open Symposia was that neither democratic educator could come up with an example of civil disobedience in democratic schools. My literature analysis revealed four types of civil disobedience: instrumental, existential, safeguarding, and expedient. The participants of my interviews in a democratic school – current teenage students, staff, and alumni – reported many instances of diverse types of civil disobedience, but primarily existential. Despite a lack of discussions of civil disobedience in the school, I discovered that the democratic school apparently promotes civil disobedience as its unintended curriculum by promoting and supporting students’ authorial agency, aiming at the students deciding what is good for them, and opposing educational paternalism.

## Introduction

Arguably, the notion of civil disobedience emerged with democratic societies at the end of the eighteenth century and the beginning of the nineteenth century. However, its practice had existed long before – the instances can be found in the Bible (e.g., the story of Mordecai and Esther) or in Ancient Greek plays (e.g., Sophocles’ play “Antigone”) (Gros, 2020). Democracy affirms the participation of every citizen in decision-making about their lives, freedoms, and rights, including the freedom of conscience. This affirmation creates tension between personal conscience and personal subordination to democratic governance. The notion of civil disobedience is one of the articulations of this inherent tension.

To my big surprise, while reading the literature on Democratic Education, I could not find any direct references to civil disobedience. Democratic schools,^1^ most famously the Summerhill school in the UK (Neill, 1960) and the Sudbury Valley School in the USA (Greenberg, 1991), commit to democratic governance of their collective affairs, like scaled-down democratic societies (Rietmulder, 2019). Yet, they do not seem to reflect and articulate the inherent tension within which this democratic governance is imbued.

My conceptual and empirical essay aims to examine the notion of civil disobedience in Democratic Education. Because of the topic’s novelty, in January 2023, I conducted three online discussions on the issue of civil disobedience with diverse international educationalists – researchers and practitioners – of Democratic Education. These online discussions deepened my thoughts on the issue. In a way, this paper was provoked by these discussions. After that, I conducted a literature review on the notion of civil disobedience in general and my attempts to find the presence of civil disobedience in democratic schools that involved the following three types of literature: 1) the literature on civil disobedience in education in general, 2) the literature on democratic education that may involve civil disobedience, and 3) the literature on civil disobedience in general. As a result of these two investigations, I conducted an empirical study of civil disobedience and self-governance in a democratic school. I included some of the feedback comments on the early drafts of the manuscript in my main text or footnotes by the participants of this study with their permission to dialogize my investigation.

## The Problem: Democratic Civil Obedience vs. Civil Disobedience

Democratic governance requires a participant’s acceptance and obedience of the democratically chosen decision, even if the participant disagrees with it, “to rule and *be ruled* in turn” (Aristotle & Jowett, 2000, italics mine). For this paper, I define democratic governance as the participants’ universal access to participation in a collective decision-making process, including deliberation, solution generation, persuasion, and politics, culminating in a legitimate imposition of the “majority” view on the “minority,” limited by the preestablished laws and constitution protecting the rights of the “minority” and other considerations. I put both terms in quotation marks because often, both majority and minority are manufactured terms that may or may not represent the actual numerical representation of the participants – they often reflect power. In representational democratic governance, citizens must accept the voting results even if their candidate lost the vote. They are also required to accept the decisions of the political body of their elected representatives, even if they disagree with these decisions. This is also true in direct democracy, where each collective decision is voted upon by the majority, and in sociocracy, where the decisions are accepted not by majority voting but by the participants’ unanimous consent. For the latter, consent is not necessarily an agreement but the expression of the participant’s acceptance of voluntary obedience to the disagreeable decision with which the participant “can live with” (Shread & Osόrio, 2018). Of course, in all its forms, democracy is heavily based on the process of public deliberation, solution generation, politics (including “dirty” one), and persuasion, which is, theoretically, although not always in practice, open to all participants, etc. Elsewhere, my colleague and I define politics as “an art of transforming power into authority—an art of how to impose your decision on people who disagree with you so that it looks legitimate to them (and, ideally, to everybody else)” (Matusov & Marjanovic-Shane, 2015, p. 202).

If a participant disagrees with a democratically made decision, several options are available in a democratic society. First, the participant can obey the decision permanently despite the disagreement. Second, the participant can obey temporarily while lobbying to change the decision through the available democratic means. Third, the participant can leave the democratic society. Fourth, the participant can *tacitly* violate the disagreeable decision and, if caught, be punished, permanently expelled from the democratic society, or jailed. The last option of being jailed does not apply to a democratic school, of course.^2^ Fifth, the participant can publicly violate the disagreeable decision as a form of public protest, hoping to attract public attention and sympathy for the cause. This can lead to democratic reconsideration, punishment of the violating participant, and/or revolt against democratic society. Sixth, the participant can initiate a revolt which, if successful, can lead to reorganization of the democratic society (e.g., rewriting a constitution). Finally, seventh, if enough people disobey, democratic society can collapse as such.

However, following legitimate orders, rules, and laws does not automatically excuse a person of moral and/or legal liability, “No one can decide for you what you can and must, and what you cannot and should not do. And a man^3^ is always responsible for what he does” (Tolstoy, 1968, p. 34) or does not do. Democratic and even some non-democratic societies do not automatically absolve a person from their moral and legal responsibility when they follow the laws, rules, and orders, even though they have been legally legitimate. Personal responsibility is not automatically excused by the person’s submission to democratically adopted decisions, orders, rules, and laws (cf. “I have no alibi in Being," Bakhtin, 1993, p. 40). Of course, probably the most famous case of the affirmation of this principle of personal responsibility is the Nuremberg trials of the Nazi war criminals of 1945–1946, where the defense of “following the orders” was dismissed by the judges. Although Nazi Germany was far from being a democratic state, this principle has been legally applied to democratic countries as well. The legal recognition of the primacy of personal responsibility over societal laws, rules, orders, norms, and traditions on morally solid grounds affirms the legitimacy of and even the necessity for civil disobedience in some cases.

## Four Types of Civil Disobedience

In his famous essay on civil disobedience, Henry David Thoreau (1849) raised the question of who the ultimate moral arbiter is: the individual conscience OR the collective norms, traditions, rules, and laws. He argued that since laws are supposed to approximate morality in a democratic society, morally grounded civil disobedience does not necessarily undermine the law but confirms it. Similarly, as the American Civil Rights leader Martin Luther King Jr. argued, civil disobedience is a display and practice of reverence for law: “Any man who breaks a law that conscience tells him is unjust and willingly accepts the penalty by staying in jail to arouse the conscience of the community on the injustice of the law is at that moment expressing the very highest respect for the law” (Luther King & Brooks, 1965, March 28).

However, in contrast to Thoreau, the American Civil Rights movement manifests the *instrumental* approach to civil disobedience as an effective and magnificent political tool for improving society. This instrumental civil disobedience is not only admired and glorified in modern culture but also currently in the *hegemonic* understanding of civil disobedience as such – it is deemed an epitome of civil disobedience. Civil disobedience is often seen as a beacon of something noble, such as the political struggle of Mahatma Gandhi, Martin Luther King Jr., or Rosa Parks for the civil rights of the Indian or Black people. However, historically, the awareness of the phenomenon of civil disobedience had emerged from a different source, namely *existential* civil disobedience as following one’s own conscience and the way of life (it is still echoed in King’s quote above).

Thus, Thoreau (1849) argued for the primacy of the personal conscience over collectivity – the person is the final judge of right and wrong. I argue that Thoreau introduced the *existential* approach to civil disobedience focusing on personal conscience as the moral compass and the only justification for civil disobedience. In essence, Thoreau unconditionally dismissed democracy based on the imposition of a disagreeable solution in favor of a personal moral judgment. His political position is libertarian. He seemed to envision a voluntary, personal anarchist society where people act as they feel morally right unless they are persuaded by others otherwise (cf. Graeber & Wengrow, 2021).

Similarly, the famous Russian writer, educationalist, philosopher, and religious and moral leader Lev (aka Leo) Tolstoy argued that the duty and the right of each person is to “liv[e] according to [their] conscience” (Tolstoy, 1968, p. 154) and dignity regardless of whether it leads to a desired societal change or not and what kind of price the person must pay for that: “standing up for your rights… as a rational and free [person], and defending them, not as the rights of local boards or committees [or government] are defended, with concessions and compromises, but without any concessions and compromises, in the only way in which moral and human dignity can be defended” (Tolstoy, 1968, p. 147). Tolstoy argued that out of all important human rights, the most important is to live according to one’s moral truth,

…all enlightened and honest people should try to be as good as they can, and not even good in all respects, but only in one; namely, in observing one of the most elementary virtues — to be honest, and not to lie, but to act and speak so that your motives should be intelligible to an affectionate seven-year-old-boy; to act so that your boy should not say, "But why, papa, did you say so-and-so, and now you do and say something quite different?" This method seems very weak, and yet I am convinced that it is this method, and this method only, that has moved humanity since the race began. Only because there were straight men, truthful and courageous, who made no concessions that infringed their dignity as men, have all those beneficent revolutions been accomplished of which mankind now has the advantage, from the abolition of torture and slavery up to liberty of speech and of conscience. Nor can this be otherwise, for what conscience (the highest forefeeling man possesses of the truth accessible to him) demands, is always, and in all respects, the activity most fruitful and most necessary for humanity at the given time (Tolstoy, 1968, p. 153).

I want to emphasize that in contrast to Martin Luther King Jr.’s and the most common modern *instrumental* understanding of the concept of civil disobedience, probably highly influenced by the civil rights movements of the 1950s-1970s, Thoreau and Tolstoy argued that the goal of (existential) civil disobedience is not necessarily to change the society or its laws, rules, or even values. Regardless of the fact that this can also be desirable, what is most important is to ensure a dignified and moral personal life at any cost, including even loss of property (Thoreau, 1849; Tolstoy, 1968), imprisonment (Thoreau, 1849; Tolstoy, 1968), torture (Tolstoy, 1968), and death (Tolstoy, 1968). According to his conviction, civil disobedience is not merely a tool for achieving desirable societal, institutional, or communal change but a way of life. Here, we see a contrast between instrumental and existential understandings of the concept of civil disobedience.

To be more exact, Thoreau (1849) seemed to argue for the pure *existential* approach to civil disobedience as a legitimate personal right, recognized by the state and society as what can be called moral libertarianism. Thoreau was not interested in improving society, “It is not a man’s duty, as a matter of course, to devote himself to the eradication of any, even the most enormous wrong; he may still properly have other concerns to engage him; but it is his duty, at least, to wash his hands of it, and, if he gives it no thought longer, not to give it practically his support. … I came into this world, not chiefly to make this a good place to live in, but to live in it, be it good or bad” (Thoreau, 1849, pp. 10, 13). Thoreau was very explicit that he was “not responsible for the successful working of the machinery of society” because he was not “a son of the [societal] engineer” (Arendt, 1972, p. 62). For example, being against the 1848 American-Mexican war, Thoreau did not want to resist pro-war American citizens – he just wanted not to be coerced to support this war by military taxation or military draft: “The only obligation which I have a right to assume is to do at any time what I think right” (Thoreau, 1849, p. 5).

In education, I found a description of such an existential approach to civil disobedience in Summerhill (without using the term). However, it was based not necessarily on following one’s conscience but on the sense of the right way of living. Michael Appleton, a houseparent at Summerhill for nine years, described an episode of Summerhill kids intentionally violating the school law of staying in at night when the lights were out in the building (Appleton, 2000, pp. 96–97). Not only did he not stop them but, after a while, he himself joined them only to observe:

…a beautiful night, well lit by the moon, and I soon traced the little band of nocturnal nomads [i.e., his Summerhill students violating the school rule] to the large beech tree that stands at the front of the school grounds, some way from the house. … Hiding behind some bushes I started to make growling noises. These were echoed by low-pitched nervous whispers and shuffling feet. Then I sprang out from my hiding place and was immediately surrounded by kids overjoyed and relieved that it was only me, and not some hideous monster that had taken up residence in the woods. For the next hour or so, I wandered around the grounds with them, exploring the shadows that the night cast, so different from the world of daylight, and charged with the thrill of being against the law (Appleton, 2000, p. 97).

The next day, Appleton (and, probably, the involved kids) was brought up to the school Tribunal by older kids, who noticed their violation of the school law and fined them. Apparently, he did not have any remorse for this violation but considered his disobedient action important because, as he put it, “I have always found such experiences useful reminders as to the child’s view of life” (p. 97). The plural word “experiences” suggests to me that he was involved in existential civil disobedience, affirming his desired way of life, which violated school laws on more than one occasion.

Summerhill was the only democratic school I know that documented cases of (existential) civil disobedience without mentioning it directly. Besides, Appleton’s book, similar cases of existential civil disobedience of principled violations of the bedtime school rules at Summerhill were illustrated and discussed in their 2008 video documentary “Imagine a school … Summerhill” (Smith, 2008, 41:55–44:15).

In contrast to Thoreau’s purely existential approach, Tolstoy argued for both *instrumental* and *existential* understanding of civil disobedience – that is why Tolstoy referred to it as a “way of living” and a “method” of societal change – as the Czech dissident and the President of the Republic, Vaslav Havel claimed: “living in truth” is “the power of powerless” (Havel & Keane, 1985; Havel & Vladislav, 1989). However, Tolstoy (and some other proponents of this approach^4^) emphasized the priority of existential over instrumental understanding of the notion of civil disobedience. Tolstoy argued for the power of the example that can sometimes produce a desired societal change. I suspect that Thoreau assumed the diversity of personal conscience, while Tolstoy believed in its universality.

Besides the instrumental value of civil disobedience as a political tool to improve society and the existential value of a personal way of responsible living, civil disobedience also has the societal value as a *safeguard* against most grievous abuses, cruelties, and atrocities by the authority in particular cases. In his short essay titled "Nikolai Palkin,^5^” Tolstoy (1968, pp. 191–200) described a disturbing phenomenon of a 95-year-old Russian petty officer who was conscience-stricken regarding the sins of terrible torturing and killing people he committed while in military service. Tolstoy's puzzlement was that the old soldier was conscience-stricken only in regard to the dreadful actions that he sanctioned himself, at his own will, but did not feel any remorse for his actions that were ordered by his superiors, which might have been as terrible, if not even much-much worse, than those that he sanctioned himself. Tolstoy argued that this abdication of personal moral responsibility under an order of authority was possible precisely because of the non-recognition of (safeguarding) civil disobedience.

Historical examples of safeguarding civil disobedience involve passionate German Nazi officers refusing to follow military orders to execute peaceful Soviet civilians in retaliation for killing German soldiers by Soviet partisans (Verhoeven, 2008). The reverse examples involved German liberals and antifascists participating in the atrocities against Jews as a part of Reserve Military Police Battalion 101 (Einsatzgruppen) while having a choice not to do that (Oldenburg & Halmburger, 2022). This issue became especially acute at the Nurenberg trials of WWII Nazi military criminals when some Nazi officers used following orders of their superiors as their excuse (cf. psychological experiments of many people’s willingness to follow deadly orders on the authority’s demands, Milgram, 1974). As a Jewish German philosopher Hannah Arendt wrote in her diary in 1967, “For centuries, men were punished for having disobeyed. At Nuremberg [trials of Nazi criminals], for the first time, men were punished for having obeyed. The repercussions of this precedent are only now beginning to make themselves felt” (Arendt, 2002). The following quote attributed to a Holocaust survivor, Primo Levi, reflects the importance of safeguarding civil disobedience, “Monsters exist, but they are far too few in number to be truly dangerous; the most dangerous monsters are ordinary men, functionaries ready to believe and obey without discussion.^6^” In education, the absence of a normalized safeguard in the form of civil disobedience costs the well-being of many students suffering from diverse severe abuses by their teachers. For example, in Russia, in an innovative (primarily progressive) environmental summer school (LESh^7^), multiple teachers sexually molested children for years (Krasilnikova, 2022, 2024).

The fourth type, which I call *expedient* civil disobedience, has been famously developed in the military, aiming at preserving the spirit of an enterprise or a task under changing, often unpredictable, circumstances. Expedient civil disobedience differs from existential one in that it focuses on a task at hand in a particular context rather than on the actor’s conscience or way of living. Surprisingly, civil disobedience, and specifically the existential type, has had a long history in the military: conscientious objectors, principled deserters, not saluting the flag, refusing to follow immoral orders, and so on. However, one particular type of civil disobedience caused by urgency, expedience, and insensitivity to the local immediate context is especially interesting because it is recognized in some armies. Thus, in one of the 2022 YouTube podcasts hosted by a Russian oppositional politician, Mark Feygin, his guest, a then-advisor of the Office of the President of Ukraine and a lieutenant colonel of the Ukrainian Army, Aleksey Arestovich, reported that one way of selecting Ukrainian officers is to give a candidate, commanding a unit, an order impossible to fulfill to see if the candidate would blindly follow this order, which unavoidably would lead to failure of the overall military operation, or take an alternate initiative, literally disobeying the order, to accomplish the assigned military task under the local conditions. According to Arestovich, this practice of recognition and even appreciation of the importance of civil disobedience in the military goes back to the Prussian Army’s practice of promoting officers’ and even rank-and-file soldiers’ initiative and instituting decentralized command and control in the pursuit of a singular military objective developed during the Franco-Prussian war (and even before, see Gunther, 2015):

… German commanders began to repeat one of Moltke’s favorite stories, of an incident observed while visiting the headquarters of Prince Frederick Charles. A [artillery] major, receiving a tongue-lashing from the Prince for a tactical blunder, offered the excuse that he had been obeying orders, and reminded the Prince that a Prussian officer was taught that an order from a superior was tantamount to an order from the King. Frederick Charles promptly responded: "His Majesty made you a major because he believed you would know when not to obey his orders." This simple story became guidance for all following generations of German officers (Dupuy, 1977, p. 116).

The story illustrated the extent to which Germans adopted mission-oriented command during Moltke’s tenure, as no less a leader than a Hohenzollern prince informed a subordinate commander that he could disobey orders when the situation called for it. This new system of command… allowed subordinate leaders independence to interpret the situation and execute actions that fulfilled the commander’s intent rather than the letter of the order.

The Prussians, and later the Germans, developed this system of command during the mid-nineteenth century Wars of German Unification. In August 1870, the Prussians defeated Louis-Napoleon Bonaparte’s Army of the Rhine in a series of battles that eventually contributed to the fall of the French Second Empire. Although French units had several material advantages over the Prussians, their commanders failed to notice a fundamental change in how the Prussians commanded and controlled their units after the 1866 Austro-Prussian War. Prussian commanders had instituted decentralized command and control in the pursuit of a singular military objective. This method of command often resulted in units entering the battle in a haphazard method during the war’s early campaigns; however, they ultimately won the battles (Gunther, 2015).

Being an officer in a Prussian army meant taking responsibility for the obedience or the disobedience of a superior’s orders. The order itself did not excuse or abdicate the officer from his responsibility.

Let me detour from the discussion of expedient civil disobedience for a moment to make more general comments about civil disobedience and personal responsibility. To paraphrase the Prussian Prince Frederick Charles in order to deepen his point a bit more, it is possible to say that *my duty as a responsible person is to make my authorial judgments about when to obey and when to disobey authority (or norm or tradition) and to take responsibility for my judgments when challenged by myself and/or others*. My judgments have to be authorial rather than formulaic, i.e., pattern-based, if I want to be the author of my own life. Thus, it is neither civil disobedience (nor civil obedience) per se that makes me unique, as some philosophers and apologists of civil disobedience claimed (e.g., Fromm, 2010; Gros, 2020). What makes me unique is my authorial judgment of when and why to obey and when and why to disobey, and me taking responsibility for my authorial judgment, which is embedded in a dialogue with others and myself.

My italicized statement above might give you a false impression that civil disobedience and civil obedience are symmetrical. This is not the case. One aspect of this asymmetry is that civil disobedience is always deliberate and, thus, eventful, while only some civil obedience is like that. Phenomenologically, my disobedience (civil or not) starts with my sense of unease that often leads to a struggle BETWEEN my loyalty to the authority and, broadly, to the external circumstances I find myself in AND my loyalty to my own perceived senses of conscience, way of living, ecology, (in)justice, dignity, sympathy to injured others, vision of the enterprise at hand, and so on (Fromm, 2010; Gros, 2020). Through this feeling of unease, I become aware of this tension. Disobedience is always dramatic, involving a collision of two loyalties. It problematizes and interrupts the flow of my life.

In contrast, only some obedience is like that. For example, in the case above, the Prussian artillery major and some, if not all, of his subordinates probably felt some unease about their bombardment of the empty field below them, seeing clearly from the hill that French troops had left the spot they were before. Some of the Prussian artillerists under the major's command might even suspect that this strange bombardment of the empty field might contribute to the failure of the Prussian attack and could cost the unnecessary loss of Prussian lives, not to talk about the useless waste of ammunition. The major and those of his subordinates might suppress their unease, perhaps justifying this suppression by professionally following orders – the military is based on the subordinates’ unconditional obedience to the superiors’ commands – or their fear of possible punishment from their superiors for considered disobedience. Thus, for them, their obedience was also deliberate, dramatic, and eventful, as in the case of disobedience.

Yet, in contrast, obedience can also be fluent, automatic, and invisible to the person. The person may not feel any unease, any doubts, or any collision about it. I may not even be aware of my obedience to authority, norm, tradition, and convention. This unaware fluency can be necessary for my life. Without this obedient easiness, the flow of my actions, my social relationships, and my communication, my life becomes impossible, which would make me paralyzed by doubts and questioning assumptions behind my actions. For instance, how can I ask a stranger for help if I do not know if the stranger speaks my language, if I do not know the stranger’s gender, age, social status, trustworthiness, intentionality (e.g., friendly, neutral, adversarial), and so on? Not only my assumptions, underlining my obedience to the existing social conventions, can be wrong – i.e., untrue – but also unjust – i.e., offensive to the stranger. If I start questioning all my obedience, my life and the lives of people around me will become like hell. Obedience is a social lubricant. Getting along – another form of obedience – makes social relationships, communication, language, conventionality, dialogue, community, and culture possible. Hence, obedience – deliberate and automatic – is both necessary and unavoidable.

However, some social lubricants of obedience can be poisonous. Some of my automatic obedience can result from being colonized by injustices such as classism, racism, ageism, homophobia, sexism, ethnocentrism, nationalism, imperialism, and so on—for which I must also take responsibility as soon as I become aware of my injustice, if not even before it. I do not have an alibi in being, which reminds me of Bakhtin (1993).

But back to the discussion of expedient civil disobedience. In education, the expedient type of civil disobedience I discussed above can manifest in civil disobedience, correcting a school’s violation of the spirit of the enterprise (for example, self-education). A few years ago, I visited a progressive private K-12 school in Maryland. A teacher gave me a tour of a newly built, bright, spacious, well-thought-out school building in the afternoon after younger students left the school. Walking over a long and broader corridor, we suddenly bumped into a child’s unfinished block construction with a note on it. The note was written by the hand of a young child. The note was signed – the author was a boy. He asked in the note not to clean the blocks because the project would be finished the next day. The teacher read the note aloud and commented to me that the school had a rule for the students to clean everything after themselves from the corridor floor by the end of the school day. The unfinished block construction was a clear violation of the school rules. The teacher was visibly perplexed with the situation. He was reasoning aloud with me (and with the boy, and with himself): “Yeah, it’s true – some learning projects may need more time to accomplish. The boy showed some responsibility by leaving the signed note and explaining his good excuse. All this is good, serving our educational mission. We support and highly encourage students to take responsibility and self-initiate learning projects. But it’s not good to leave a pile of blocks in the middle of a corridor for a day. It is hazardous and may create a license for chaos. This time, we may want to overlook his transgression, but I’ll talk with him tomorrow.” “Maybe you should consider changing the rule?" – I suggested.” “Maybe,” replied he, but his voice reflected uncertainty if not improbability. The faculty created the overall rules in this progressive school at a faculty meeting and not through democratic governance with the students. I did not know if the teacher’s discretion of overlooking the boy’s transgression and not reporting it was legit – i.e., allowed by the faculty – or if it was another occasion of (expedient? existential?, both?) civil disobedience, this time by the teacher who was not going to punish the boy, in addition to the boy’s civil disobedience of the school rule. However, it was clear to both of us that the boy’s civil disobedience was caused by the school rule’s violation of the spirit of the progressive school’s enterprise to promote long-term projects that the faculty viewed as having a high educational value.

## Civil Obedience vs. Civil Disobedience

On moral grounds, Thoreau unconditionally dismissed democratic (or any other) obedience in favor of (existential) civil disobedience. However, not only the law or the authority order can be corrupt, wrong, insensitive, or ineffective. It is also possible for a personal moral judgment to be embedded in oppressive communal values such as racial, homophobic, xenophobic, class-based, and misogynistic bigotries. Corruption is an authorial judgment by an observer – it is in the eye of the beholder, which again brings us back to collectivity and its discourse, where this judgment is made. For example, in September 2015, driven by her Apostolic Christian principled homophobia and conservative political conviction, Rowan County clerk in Kentucky, Kim Davis, denied same-sex marriage licenses and, thus, violated the law of the land, affirmed by the Supreme Court of the United States on June 26, 2015. She abused her legal duty and power as county clerk. She disobeyed the judge, for which she proudly went to jail^8^ guided by her conscientious conviction and paid $260,000 in legal fees and damages to a gay couple whom she denied a license.^9^ Arguably, this was a case of *corrupt* civil disobedience (both instrumental and existential). What makes it corrupt in the eye of the beholder is a judgment of the legitimacy of Davis’s conscience.

Thoreau unconditionally prioritized civil disobedience over obedience to societal norms, rules, laws, and traditions (i.e., extreme libertarianism). Counter to Thoreau, I found the opposite universal resolution of the inherent tension between democratic governance and civil disobedience: namely, this alternative approach dismisses civil disobedience (i.e., extreme democratism). In this alternative approach, the resolution of the tension between democratic governance and personal responsibility claims that a well-designed open democracy does not require civil disobedience at all because all disagreements can be resolved through democratic means, primarily through rational persuasion (Anna Danilova, the Third Open Symposium for Russian democratic educationalists, January 20, 2023). For example, at the First Open Symposium, the American scholar and practitioner of Democratic Education, Melissa Bradford, remembered reading a case presented in Danny Greenberg’s essay (a co-founder of the Sudbury Valley School). In his essay, according to Bradford, Greenberg described:

…[it was about] a block of students at the Sudbury Valley School who were discontented. They felt like their voices weren’t heard [at a school assembly]. Danny [Greenberg] would talk with them about it. He would say, ‘Well, come to the school meeting. You have to make a good argument.’ And they did. Unfortunately, they felt that they had done that, but it didn’t work [they could not convince the rest of the school]. And so, the impression I got [from Greenberg’s essay] is sort of like you have to be persuasive enough: you’ve got to come in here [i.e., school meeting or committee meeting], and you’ve got to convince people to do things the way you want them to do. Otherwise, it’s on you; it’s your personal responsibility^10^ (Melissa Bradford, The First Open Symposium for American democratic educationalists, January 3, 2023).

I found a similar belief in a good democratic governance system that can resolve all injustices through its systemic democratic governance in A. S. Neill’s writings about his famous Summerhill School in the UK. When asked about what would happen when a Summerhill student refuses to pay the fine imposed by a General School Meeting, Neill replied, “Children never do. But I expect they would refuse if they felt they had been treated unjustly. Our appeal system overcomes any sense of injustice” (Vaughan et al., 2006, p. 139). Similarly, a philosopher of self-governing education, Ronald Swartz argued that in a tolerant, liberal, society with reasonable laws, civil obedience is the must: “Of course, this third recommendation [i.e., total civil obedience to democratically established rules and laws] only holds for societies which one considers to be tolerable; for those who find themselves living under unreasonable laws, civil disobedience may be justified” (Swartz, 2016, p. 145).

This universalist approach seems to be based on the conviction that any disagreement can be resolved through a sophisticated rational discourse, which will lead to a consensus,^11^ consent, or at least a majority agreement (Habermas, 1984).^12^ In my view, this rationalist approach is somewhat questionable and infeasible because there can be diverse and irreconcilable rationalities based on diverse and irreconcilable values (Kelly, 1978; Matusov, 2021).

Finally, civil disobedience and civil obedience can create the Gordian knot when it is super difficult to separate one from another. For example, in the United States, several educationalists have noticed the phenomenon of “Acting White,” where some African American students undermine other African American students for doing institutionally well in school, which they see as a White institution of historical and present racial oppression (Carbado & Gulati, 2013; Fordham & Ogbu, 1986; Ogbu, 2003). Civil obedience to the particular peer and community culture becomes equal to civil disobedience to the school institution and vice versa. Separating obedience vs. disobedience requires an authorial value-judgment perspective of what constitutes a good norm.

In sum, I do not think that any universal, decontextualized principle -- e.g., extreme libertarianism, extreme democratism -- can resolve the tension between democratic obedience and civil disobedience in favor of one or the other. Paraphrasing Howard Zinn, both civil obedience and civil disobedience can be problematic.^13^

## Definition: What is Civil Disobedience?

Some literature on civil disobedience equates “civil” with peace and non-violence (e.g., Moraro, 2007; Morreall, 1976). I think it is partially true. The Latin-origin word “civil,” like the Greek-origin word “polite,” has the etymology of living in a city (in Latin, or polis in Greek). A city is a peaceful, dense, mutually beneficial, voluntary coexistence of “tribes/communities” and “strangers,” often with incompatible diverse values and cultures (Graeber & Wengrow, 2021; Matusov, 2024a). Civil disobedience arises in the context of a person’s confrontation with the power of peers, groups, families, institutions, communities, the public, or the state in the “city.” Democracy (in a broad sense) is the legitimacy of participation in the discussion and adoption of collective and private decisions about the fate of each member of the "city." Thus, civil disobedience is a deliberate peaceful non-cooperation, protest, or violation of a legally (democratically) established rule/decision/order/law/tradition/norm due to disagreement with it. The issue of whether these non-cooperations, protests, disruptions, or violations and their consequences are truly peaceful is always in the eye of the beholder. For example, protesters blocking road traffic may see themselves as peaceful, while from the point of view of a husband who drives his pregnant wife, whose labor has started, to a hospital, the protesters’ actions might be seen as violent.

Besides being peaceful, for disobedience to be “civil,” it must be *principled* (Jim Rietmulder, the Second Open Symposium for American democratic educationalists, January 13, 2023). This requirement contrasts with disobedience caused by a person’s impulsivity, immaturity, ignorance, and/or insensitivity. For example, in the democratic school I studied (see below), a group of teenagers used foul language in a public space, which violated the school’s democratically accepted rule of not using foul language in public spaces. Jim Rietmulder, a founder of the school, did not consider this public disobedience of the school rule “civil” because it was not principled in his view. Jim Rietmulder argued that the teens did not have any ideological principles to object to the anti-vulgarity rule in public spaces. The teens might forget about the rule at the moment and/or might be insensitive about their surroundings during their intense, emotionally charged discussions (Jim Rietmulder, the Second Open Symposium for American democratic educationalists, January 13, 2023). Although without talking with the teens involved in the incident, we cannot know this for sure, Jim Rietmulder’s suspicion about the teen’s disobedience being unprincipled sounds plausible to me. I agree that non-principled disobedience does not constitute civil disobedience. For example, some teachers worry that certain participants in the Fridays For Future international protests organized by the 15-year-old Swedish activist Greta Thunberg – weekly skipping school to protest global ecological causes – might be driven by peer pressure, fashion, or dislike of school in general rather than genuine personal concerns about global ecology.^14^

Another case of non-principled disobedience involves a clash of cultures and cultural expectations. During our Third Open Symposium on Civil Disobedience, this time with Russian democratic educators, Aleksey Shirshov reported an incident of his participation in Russia in a workshop about Summerhill School led by Henry Readhead (Third Open Symposium on Civil Disobedience, January 24, 2023). Henry organized a group that was supposed to simulate the work of a Summerhill democratic school committee to collectively solve a concrete (imaginary) problem: what to do with garbage – a simple issue to vote on after deliberation. Aleksey’s group was sitting at a round table and discussing the issue. For Aleksey, the communicative norm was “lively dialogue” in which people interrupted each other when they talked too much, from their point of view. He started doing that as he acted in life. After a while, to his surprise, Aleksey was expelled from the group as “a bad guy” because he did not follow the group’s communication norms and expectations. He did not follow clearly defined speaking turns and tried to transcend the group’s technical discussion by moving it to more philosophical issues. Aleksey told us that he had not tried to deliberately disobey the group’s norms, not tried to be “a troublemaker,” but instead attempted to achieve the set goal of the group as he had understood it. According to Aleksey Shirshov, the moratorium on the philosophical discussions and clear-cut dialogic turns was not explicitly established by the group (or by the leader of the workshop, Henry Redhead). Although the group and the leader of the workshop qualified Aleksey’s behavior as disobedience, I would not qualify his behavior as such. And, in my view, Aleksey was definitely not involved in it as civil disobedience at that moment but rather in organizational and, probably, cultural misunderstanding. However, later, he might reflect on the differences between the group’s and his own cultural communicative norms and goals of the activity and elevate these differences into principled ones, refusing to accept the moratorium on philosophical discussions in principle.

Similarly, I would not consider a case of a “happy system crasher” (Matusov, 2024b) as civil disobedience. I described my own first-grade student experiences. I lived my own happy life, which contradicted the school’s institutional life. My teachers and parents might interpret my behavior as resistance or civil disobedience, but that was not my interpretation at the time. I tried to be a good student. However, my definition of being a good student contradicted the school’s. As in the case of Aleksey Shirshov, discussed above, it was more like a cultural misunderstanding. However, like in his case, existential civil disobedience crystallized from this cultural misunderstanding when I fully realized this cultural disagreement and chose “my side” as a principled way of living. For example, throughout my school career, I systematically crossed out the official textbook answer if it contradicted my answer after I checked my answer again and again. I was often punished by some of my teachers for this existential civil disobedience.

A principled disobedience that can constitute civil disobedience involves the person’s deliberate ideological objection – a principle, a conceptualization – against the law, rule, order, norm, or tradition established by the authority. At our Second Open Symposium on Civil Disobedience at The Circle School, an American democratic private school, on January 13, 2023, a disagreement emerged about how verbal, explicit, and articulate an objection must be considered “principled.” This issue is especially important for civil disobedience by very young children, who might not have the ability or inclination to verbalize their ideology conflicting with the official one.^15^ Bryan Greenberg, a friend of The Circle School who attended the Symposium, shared the following autobiographical story. When he was 5, he was sent to a day camp by his parents:

We all had to change into bathing suits in a big room. I did not like that. There were our counselors: women and girls. I was uncomfortable with that. I felt in whatever way that this was wrong; this rule was wrong. I refused to change. It created a bit of a ruckus until they finally allowed me to go off to the bathroom to change. I went off to the bathroom to change. Within a few moments, other boys started coming into the bathroom. And so, my lack of principles, maybe I wasn’t doing it to change the world, but I think I took a stand that led others to affirm their desires and sensibilities (Bryan Greenberg, The Second Open Symposium for American Educationalists, January 13, 2023).

Was Little Bryan’s disobedience to change in public with women and girls principled or not? Jim Rietmulder, a school co-founder, objected: “I cannot believe that at five years old, or I’m reluctant to believe, that at five years old child is going to conceptualize it as either civil disobedience or any kind of moral or principled objection.” Similarly, at our Third Open Symposium on Civil Disobedience with Russian democratic educators on January 20, 2023, Aleksey Shirshov expressed apparently similar ideas, referring to a famous Russian philosopher and “methodologist” Petr Shchedrovitsky.^16^ According to Shirshov, for disobedience to be civil, it must have “a cultural written text” justifying the person’s objection to a rule, law, norm, order, or tradition. Without such cultural written text, disobedience is a caprice not to be taken seriously by others.

The rest of the Second Symposium participants at The Circle School, including me, disagreed with Jim Rietmulder (and, thus, with Shchedrovitsky in Shershov’s presentation). Although I concurred with Jim that a five-year-old child usually cannot articulate a verbal argument (e.g., “public changing in the presence of women and girls violates my male privacy and dignity”), objecting to a rule, a young child can conceptualize through their emotions, “I *felt*^17^ in whatever way that this was wrong; this rule was wrong” (see Bryan’s account above; italics is mine). A child might feel a principle through their emotional conceptualization (rather than verbal argumentative conceptualization). Morality – what is right and what is wrong – can be deeply felt. Bryan concurred with me: “There has to be some conceptualization. Isn’t there some conceptualization just on the basis of… that I have to be aware that I’m uncomfortable and it’s not right to me? It might be a more rudimentary concept or conceptualization, but it’s there.” Thus, conceptualization can be verbalized, but it also can be emotionalized through actions, behaviors, gestures, and facial expressions – in both cases, conceptualization becomes communicated to the self and others.

However, Jim still rejected our arguments at the meeting, insisting that the principled objection requires the person to have a desire to change the world rather than to address personal discomfort: “Yes, it is. But it’s not a conceptualization of civil disobedience. It’s just much more emotional and visceral than thinking about society and the rules.” Although Little Bryan’s disobedience led to changing “the world” – other boys who felt the same way as Little Bryan were allowed to change privately in a bathroom, Jim Rietmulder was right that it was *not* Little Bryan’s primary intention.

Should the definition of civil disobedience (of any form – verbal or emotional) require its actor to have an intention “to change the world” (i.e., society, institution, community) to be civil disobedience? Using the Hegelian philosophical terminology, should the objecting principle be only the *universal* (i.e., for all and always) or, at least, be the *particular* (i.e., for some and sometimes)? Or can it also be the *unique* (i.e., for me and now)? I think the latter, which is especially true for existential civil disobedience (but also can be applied to the other types). Like in the case of Little Bryan, I argue that the universal or particular civil disobedience often starts with a *unique* principled objection for one unique person here and now, and then it can spread to others across times and places and, thus, become particular or even universal (e.g., other boys went to bathroom to change). In the first civil disobedience, the given culture gets transcended by the person who objects to the existing rule, law, norm, order, or tradition. A possibility for a new “cultural text” emerges with a person’s creative transcendence of the previously given cultural texts, which may or may not be done through civil disobedience. Thus, I argue that the definition of civil disobedience does not require its actor to have an intention “to change the world.^18^” As I discussed above, instrumental civil disobedience as a political tool for the improvement of society is only one of many types of civil disobedience. The case Little of Bryan is a clear example of existential civil disobedience rooted in the violation of personal dignity perceived by the 5-year-old boy.

Another definitional issue for the concept of civil disobedience is whether the person’s acceptance of possible punishment in advance is the necessary part of the definition. As Martin Luther King Jr. stated in his quote above, civil disobedience is associated with willingness to accept “the penalty by staying in jail…” Is the person’s willingness to suffer a necessary component of what makes disobedience civil? This willingness may affirm two opposite convictions of the actor of civil disobedience: 1) a strong conviction of the actor’s righteousness even in the face of a possible severe punishment (even death) and 2) the actor’s acceptance of the authority’s final judgment about the civil disobedience and the rule, law, order, norm, or tradition when punishment is one of the possible consequences of such judgment. Of course, an actor’s willingness to accept possible punishment for their civil disobedience – in the extreme, the willingness to sacrifice their life – can be viewed as an indicator of the actor’s stronger civic position, conviction, and commitment in a comparison with another actor who is not willing to accept possible punishment in advance of their civil disobedience. Although I agree that willingness to accept the risk of a possible punishment clearly reflects the principled nature of disobedience, the reverse is not necessarily true. Namely, I question the reasoning that the absence of the person’s acceptance of punishment signals that disobedience is not principled. The person might not accept the punishment or even risk the punishment because they might not see themselves as guilty (Arendt, 1972).

Finally, I want to discuss the issue of fake civil disobedience in education. Probably, the biggest fake civil disobedience in education is to use students for the act of the educator’s social justice. In my view, one of the most outrageous cases of fake civil disobedience was the *Kinderläden* movement in 1970s West Germany. To address the historical legacy of German people’s obedience to the Nazi regime, left-leaning parents created innovative kindergartens with the so-called “curriculum for disobedience” (Appelbaum & Davis, 2013). For example, the adults in *Kinderläden* brought very young children to public spaces to disrupt the public order and conventional norms. Apparently, being upset with the negative publicity of the *Kinderläden* movement, the adults at the *Kinderläden* “collected used diapers from the city’s Kinderläden for three days. Then, children and adults carried the diapers in garbage bags back to the Press Office, entered the newly painted chambers, and smeared the walls with the diapers’ contents to express their displeasure with those who had control of voice and opinion in the public sphere. With such an act, adults fought against their own deeply ingrained habits of authority and propriety; they sought to demonstrate the same to their children” (Appelbaum & Davis, 2013, p. 150). To teach resistance to authority, the Kinderläden educators encouraged little children to get into adults’ underwear and pull their pubic hair. Amazingly, these educators did not notice that they imposed their own authority on the children – it was the children’s obedience to adult civil disobedience. Arguably, the adults hijacked the children’s agency for their own adult political causes. In my judgment, this made Kinderläden’s civil disobedience fake.

There is well-known global political student civil disobedience involving the students’ walk-out their classes, protests, blocking entrances to the classrooms or schools, occupying campuses, boycotting, strikes, and so on. The global political causes can be very different: anti-war movements, demands of certain actions or changes by the government, solidarity with political prisoners, supporting Hamas, an internationally recognized terrorist organization, protesting against global warming or population growth, etc. Some of such student disobedience is not civil; some are fake – i.e., the students’ civil disobedience is a result of being brainwashed or pressured by peers, parents, teachers, political organizations, and/or even the government. Some are real, being principled by the students’ own deep convictions. In the latter case, student civil disobedience can be purely instrumental, focusing on improving society, or a mixture of instrumental, existential, and/or safeguarding. Students can be punished for this political civil disobedience by lowering their grades, administrative pressures, expulsions, arrests, and so on.

Student academic civil disobedience can involve academic, education-related, and non-academic causes. Thus, for example, in the 1960s, American students demanded diversification of the official curricula through including minority studies (Duberman, 1969). In a fictional Soviet movie, “We’ll live till Monday,” high school students walked out of an English class because they protested the novice English female teacher’s harsh treatment of them, being “nice” to them before (Rostotsky & Polonsky, 1968). In the Soviet Union, many Estonian, Latvian, and Lithuanian high students rejected the Soviet official history, teaching that these Baltic republics voluntarily joined the Soviet Union in the summer of 1940 and were not a result of the brutal Soviet occupation. These students could spit back the “historical facts” during the history lessons and exams but privately and personally rejected them. This type of student resistance – a form of existential civil disobedience – was called “internal emigration” (Wertsch, 1991).

Teachers can also participate in diverse forms of political and academic civil disobedience. For example, being a Soviet physics teacher, I sabotaged teaching my students official Soviet propaganda while it was a part of my official teacher responsibility (Matusov, 2018b). I judge this form of teacher civil disobedience as existential, driven by my convictions about what it means to be a professional educator who must not be involved in any imposition of dogmatism, brainwashing, and political propaganda, regardless of the underlying values. Similarly, in my university teaching in the USA, I sabotaged forcing my students to accept progressive leftist social justice values of the day, regardless of how much I might agree with them (Matusov & Lemke, 2015; Matusov, 2018a).

In sum, *I define “civil disobedience” as a personal principled conviction, expressed verbally and/or emotionally, of wrongness or evilness of the established order, rule, tradition, norm, or law imposed on the person and/or other people and occurring on a systematic basis for many people or here and now only for one unique person, that leads the person to peacefully violate this order, rule, tradition, norm, or law*.

Now, I am turning to an empirical investigation of the phenomenon of civil disobedience in a democratic school.

## Research on Civil Disobedience in a Democratic School

The purpose of this research was to explore the paradox of democratic governance in education. On the one hand, democracy is based on the participants’ voluntary subordination to the rule of the majority (however, it might be limited by a constitution and previously democratically established laws) even when they disagree with this rule.^19^ On the other hand, democratic governance in itself (i.e., following democratically elected rules) does not automatically excuse people from their moral, educational, relational, self-actualization, and other responsibilities to themselves and others for their own deeds (or a lack of them). Specifically, I wanted to examine the presence, nature, definition, necessity, and place of Civil Disobedience within a K-12 democratic school. For this research, I operationalized the concept of Civil Disobedience as people’s particular response to school rules that they do not like (i.e., a “bad” school rule) but cannot change. This response involves non-violent resistance to the disliked rule.

Definitions:

Democratic school is based on self-education when education, curriculum, and instruction are not imposed on the students: when students make their own decision whether to study, what to study, with whom, how, when, and so on, with or without the help of other people. Also, the school participants (the students and the staff) are involved in democratic governance of the school.^20^

The particular site of my research was The Circle School^21^ (TCS) that was established 40 years ago. I have been familiar with the school for 14 years, being a friend of the school and a close colleague of the school’s founder, Jim Rietmulder. It is a small private school with 42 students, ranging from 4 to 18 years old, and 5 staff members at the time of research. The school is located near Harrisburg in a gorgeous and well-thought-out building constructed recently for the school on a picturesque site surrounded by woods and a creek.

Civil Disobedience is a principled and peaceful refusal to submit to a democratically accepted school rule or norm. It may involve a peaceful violation, protest, or sabotage – public and private, hidden or spectacular – of a school rule or norm when the person feels it violates their or other people’s morality, dignity, basic needs, well-being, self-realization, education, purpose, spirit, etc.

Research questions:

1. Is civil disobedience present at the TCS in the perception of its participants? If so, why? What are the reasons and nature of civil disobedience there? What definition of civil disobedience emerges from the data? If not, why not?
2. Do TCS participants recognize the necessity for civil disobedience? Or not? Why and why not? Is civil disobedience a “hidden,” better to say unintended, curriculum^22^ at the school?
3. Do TCS participants feel that the democratic school adequately recognizes and addresses cases of civil disobedience? Or not? Why and why not? What are their recommendations for improvement?

The main research method involved interviews with current students, staff, and alumni. I enrolled the participants via a survey, meeting with them at the school, and “a snowball effect” when a volunteer participant brought their friends or colleagues to the research. It is important to acknowledge that despite its effectiveness, the snowball enrollment of research participants might create a possibility of bias by following only like-minded participants at the expense of the diversity of existing views.

I interviewed seven teenage students (four females and three males), three new staff (all of whom are school alumni), two senior staff, and one alumnus who is not a staff member, who volunteered to participate in my research. The interviews with the students were one-on-one, except for two dyad interviews. The old-time staff were interviewed together. Two novice staff members were also interviewed together. One novice staff member was interviewed separately. All these interview choices were made by the research participants. All interviews but one were face-to-face. The school alumnus, who is not a staff member, was interviewed via Zoom. All interviews were recorded with the permission of the participants.

My analysis was primarily qualitative, focusing on abstracting patterns and making sense of them. It was not aimed at generalizing about democratic education as such, other democratic schools, or even this particular democratic school. The interviews focused on the school rules that the interviewees disliked (referred to in my interview as “a ‘bad’ school rule”), the reasons for the dislike, and the responses to that. Also, I asked the interviewees about the desired approaches to the disliked school rules (i.e., their normativity). The interviews were openly structured and often led to discussions of problems with the democratic governance process in the school (not included in this paper). The presented quotes might be slightly edited for comprehension. The previous completely anonymized drafts were shared with the research participants for accuracy, feedback, value disagreements, and safety concerns. Some participants provided holistic feedback, some added comments, and some corrected, edited, or clarified their quotes. All substantive feedback responses were included in the report either in the main text of my research report or as footnotes. Some participants (two students, one junior staff member, and one senior staff member) decided not to provide any feedback, allowing me to use their quotes as they were. Based on the school’s staff members’ decision, all children’s names were anonymized, regardless of the children’s wishes. Some adult participants wanted to use their real names in the paper, and I followed their wishes. Alternatively, I used pseudonyms. The staff insisted on making the school known for publication. The interviews were conducted in the winter-spring 2024.

TCS interviewed students (all students’ names are pseudonyms):

**Table:** 

Name	Age	Years at TCS (as of June 2024)	Type of school before TCS
Abigail	16	2	Conventional
Pam	16	5	Democratic
Sally	16	2	Conventional
Nathan	13	9	TCS all the time
Linda	15	4	Conventional
Alan	15	1.5	Progressive
Steve	14	5	Democratic

TCS interviewed junior staff (* pseudonym):

**Table:** 

Name	Student years spent at TCS	Staff years spent at TCS
Samantha*	5	2
Cody	5	3
Brook^23^	4	3/8

TCS interviewed senior staff^24^ (* pseudonym):

**Table:** 

Name	Staff years spent at TCS
Beau*	40
Jim Rietmulder	40

### Research ethics

The research was approved by the school’s research review board. In my research, I prioritized not putting the participants in any danger by revealing their violations of school rules to other school members. Because of that, I omitted to present some of my findings, which did not change my conclusions.

### Findings

The main finding is the participants’ numerous reports about instances of existential civil disobedience, which suggests to me that the school promotes a sense of human dignity in its students. The cases of instrumental and expedient civil disobedience were also reported, although they were much fewer than existential ones. The safeguarding type of civil disobedience was not reported. I did not find any other (new) forms of civil disobedience.

The findings indicate a strong condition for and evidence of civil disobedience at the school. By the strong condition for civil disobedience, I mean the students’ perception of “bad”^25^ rules and norms at the school that they were not able to change through the school’s democratic governance process. Democratic governance alone cannot appear to address all cases involving “bad” school rules.

At the same time, I found evidence of an unintended, byproductive curriculum that promotes existential civil disobedience in TCS students through practicing their authorial agency.

#### Evidence of diverse types of Civil Disobedience at the school

The seven interviewed teenage students reported altogether ten “bad” rules and norms, five of the rules they tried unsuccessfully to change through the school’s democratic governance. Several students who did not try to change the school rules they thought to be “bad” reported that they were discouraged by observing how their peers were unsuccessful in trying to change theirs. One such student was part of a group that was trying to change a “bad” rule of always having lights on in school during the school day, which violates a sense of privacy for teenagers (see below): “We had it all planned out. We took it to a school meeting. And school meeting did not take it very well” (Linda, student, Interview).

Some of these “bad” school rules affected only individual students. For example, the school meeting secretary, Abigail, a novice TCS student, wanted to have a new school rule requiring submitting new agenda items and reports at least 24 hours prior to the upcoming school meeting unless it was an emergency. The concurrent school rule allowed the submission of amendments at noon on the day before the school meeting. According to the student, this “bad” school rule made her work less manageable and her life more stressful. Another “bad” school rule affected many, if not all, participating students, was keeping all building lights on (see it in the School Rulebook in Appendix). Teenage students complained about this rule because they felt it violated their privacy and created a surveillance panopticon effect (cf. Foucault, 1995) in addition to numerous windows in the building (moreover to energy waste). They argued that the lack of privacy affected all people in the school, especially adolescents, for whom privacy was more important than for younger students or adult staff. Some “bad” school rules sounded “absurd” to the interviewed students – for instance, a rule unconditionally requiring whispering in the library. The rule sounded ridiculous to the students because there might be no people in the library who would require a quiet space (see the school rule in the Appendix).

One “bad” school rule forbidding sitting or lying on the school’s counters was successfully changed “via a compromise” with a staff member. The school meeting voted on the rule changes. But, according to the author of the proposal, Pam, for a new rule, the success was very limited while the cost of this change was prohibitively high,

Well, I do think that it was successful in the sense that, you know, the motion passed, and it’s there now. But I think for all the effort and time and emotional energy that went into passing the rule that it’s not enough of a change to have been worth it.^26^ I think if we could have made that same change happen in just one meeting where everybody was like, you know, all in agreement, it [would have been] fine. It would’ve been worth it. I would think more optimistically about it, but because it was just hours and hours and hours and hard, it doesn’t seem like the cost. … It [took] a month and a half, but only maybe four total meetings. … And so after the meeting, there was a lot of people like who were upset by it and there was hostility. It felt like it damaged my relationships with a couple of people who were disagreeing with it. And that those are only just starting to recover. It’s staff. It’s the relationships of mine that were damaged with staff because it was only staff members who voted against the motion or who spoke against the motion. Only two staff members actually voted against it. But somehow, with just three of them, they managed to make the conversation go on for so long (Pam, student, Interview).

The teens’ most typical response to “bad” rules, which they could not change, was to obey the rules, not to protest them:

…my strategy, if I don’t agree with the rule to such a degree that I wanna change it I definitely try that first. 99% of the time, it doesn’t work. But I will fight for as long as I feel like I can fight until I get so discouraged from the constant wearing down of school meetings that I give up. And then, usually, I end up just keeping my head down and following the rule because I don’t wanna get in trouble. I want that clean record. I don’t want to be in JC [Judicial Committee] every other day, you know, defending myself. I don’t. So, just keep my head down, I guess. I’m not gonna get in trouble. …

I feel as if I have a strong fear of rule-breaking because of my time in [conventional] public school [prior to this democratic school]. I do think that the more I am at The Circle School, the more comfortable I am with the idea of opposition [to “bad” rules]. I wonder how I would feel about rule-breaking if I had been going since I was young^27^ (Abigail, novice student, Feedback).

I don’t actively try to break [bad] rules^28^ like ever as a form of protest because … I don’t think it would have an effect. I don’t think it would matter that much. Or I think that me doing it as a protest would get me in a lot more trouble than the rule itself, like breaking the rule itself (Steve, Interview).

However, a few individual students reported intentional violations of the “bad” rules, including the ones described above, because they were making sure that nobody could see them violating a “bad” rule and they would not get in trouble.^29^ I qualify these instances as an example of a particular type of civil disobedience. Below, I provide unidentifiable cases of principled violations of the “bad” rules.

Initially, all interviewees understood the notion of civil disobedience as active public actions of protest to change disliked rules, i.e., the instrumental approach. This understanding did not fit their covert actions of private disobedience, “I don’t really know that I would call it civil disobedience because I’m not trying to enact [a] systematic change. I’m just trying to work the system” (Pam, student, Interview).

As far as the interviewees remember, there was only one such “classical” instrumental civil disobedience case of a protest aiming at a societal change as remembered by the staff members – a senior (Beau) and junior staff member, Samantha (when she was a student back then), – in 2007. Beau told me that a female teenage student decided to protest the societal dress sexism and, to a lesser extent, to start the conversation and eventually change society by addressing its sexism. Specifically, she was upset that for men and boys, it was okay to present themselves topless in public places, beyond beaches, but not for women and girls. So, she decided to protest by taking her T-shirt off while leaving her bra on at a place of school gathering. Beau (senior staff) noticed this protest and talked with the protesting student, trying to dissuade her from this transgression of the school rules by arguing that 1) this sexism was not much of a problem at the school and 2) it might hurt the school because female toplessness contradicts local (and national) communal roles, sensibilities, and expectations at that time.^30^ The protesting student seemed to get persuaded and put her T-shirt on. Beau did not write up the student transgressing the school rules to refer her to the school’s Judicial Committee (JC) because, according to Beau, the female student obeyed after the warning. In my conversation, Beau condemned the female student, implying some cowardice, hypocrisy, and immaturity on her behalf because the school was the safest, most supportive, and cozy environment that did not need such a protest. Further, according to Beau, the student’s protest was non-influential because apparently nobody even noticed this event except Beau. However, a junior staff member, Samantha, who was a teenage student back then, reported that she and her friends noticed this event and discussed it with each other, considering it silly. Samantha added, “It just didn’t really feel like there was a point to that. Yeah. I would personally say that that’s probably the way that happened was influenced by her family and the way she was raised” (Samantha, Interview). Samantha seemed to imply that the girl demonstrated a *fake* political instrumental civil disobedience, being heavily influenced, if not brainwashed, by her liberal family rather than being principled in her disagreement with sexist society. This might or might not be true. When I asked Beau and Samantha why the protesting female student was not supported in one way or another since the democratic school committed to promoting the students to “practice life” (Rietmulder, 2019), they seemed visibly perplexed by this apparent contradiction.

This case raises the issue of finding a contextual balance^31^ between the safety and survival of the school and its declared mission of letting the student “practice life.” I wonder if many of the staff’s decisions have been skewed^32^ toward the school’s safety and survival at the expense of the students’ practicing life,^33^ which promotes their conservativism, which was noticed and discussed by the students in the interviews. But, of course, I could be wrong because the school staff might be strategic in “choosing their battles,” so to speak while protecting and defending their educational radicalism at the expense of their civic liberalism. I hope to spark future discussions at the school about this issue of seeking the balance between the school’s safety and school mission,^34^ as well as building a transparent organizational mechanism for finding this balance. Abigail (student) commented in her feedback on this paragraph: “I totally agree with this section. Oftentimes, it is frustrating when the future comes before the present.”

Both the interviewed staff and students seemed not to be familiar with non-instrumental types of civil disobedience that do not focus on changing society (“Definitely, agree with that,” Abigail, student, feedback). However, when I introduced Thoreau’s and Tolstoy’s notions of civil disobedience as living according to one’s consciousness or in peace with oneself, the existential approach, the interviewed students seemed to like that as well,

…in general, I think that you should live by your morals. Right? I think I agree with that part of it. You are who you are, and if you break a minor law every here and there, as long as you’re enjoying your life at the end of the day, then that is what is most important (Abigail, student, Interview).

I think [civil disobedience as living according to one’s conscience and at peace with oneself] is much more the attitude that I have adopted. I’m not trying to change it [i.e., a "bad" school rule], but I am a little bit trying to circumvent it. … I would agree with that. I think that the importance of trying to make the actual change is in the way that I’m getting away with breaking these rules because of the type of privilege that I have at this school. And that is, I think, very easily applicable to the outside world. I could probably circumvent many things about the world that I take issue with. Whereas someone with less privilege who is just assumed to be breaking the rules is more likely to get caught. So, I would say, you know, it’s maybe more morally upstanding to actually try to make the change as opposed to just ignoring the rule. But where I’m at, I’m just ignoring the rule^35^ (Pam, student, Interview).

I was not sure if some of these students had changed their minds about their approach to defining civil disobedience after our interviews. Still, at least, they seemed to have considered broadening their understanding of civil disobedience. Abigail (student) commented on this statement in her feedback: “It was definitely an interesting conversation that made me consider what Civil disobedience was.”

There are several cases of reported existential civil disobedience, such as the promotion of students’ ways of life that contradict the school rules. Thus, to promote their privacy at the school, teenage students dimmed down the school lights in some school rooms, where teenagers seeking privacy gathered. Formally, *de jure*, by dimming the light all the way down, the school rule of keeping the lights always on around the building during the school days was observed, but in reality, de facto, it was violated^36^ – a civil disobedience strategy of following the letter of a rule while violating its spirit that Soviet dissidents used in Soviet prisons (Bukovsky, 1979). Interestingly, although Beau (senior staff) noticed a dimmer switcher pulling all the way down in one of the school rooms, she did not think that “they [i.e., teenage students] ever went into a room and just deliberately turned the lights off knowing that they shouldn’t” (Beau, Interview). However, the other senior staff member, Jim, somewhat disagreed with Beau. He thought that dimming the light was deliberate but did not constitute civil disobedience, “It was deliberate during the weeks that the lights-on rule was being debated. At the time, I understood some of it to be experimental and some of it to be exercising their right to do it while they understood it to still be lawful or while we were trying to figure out if it WAS lawful. Other times, it was just that someone wanted the lights to be dim” (Jim Rietmulder, senior staff, Interview). Some of the interviewed teenagers mentioned that they did it deliberately and subversively as a part of their principled disobedience of the light school rule because it violated their sense of privacy, which is so important for young people.

Throughout my interviews and conversations with senior (but not junior!) staff members,^37^ they mostly denied, dismissed, or downplayed cases of civil disobedience at the school. One of the reasons for that was probably the hegemonic instrumental understanding of civil disobedience as a political tool for changing society. A senior staff member, Jim, commented,

Of course, the school has abundant “preconditions for civil disobedience.” The school is not a utopia and wouldn’t fulfill its promise of “practicing life” if it aimed to be so.

Life with human beings is messy. I hope our school makes space for students to experience “negative” behaviors (such as dishonesty, theft, rudeness, vulgarity, and, yes, civil disobedience) and also “positive” behaviors (such as honesty, kindness, fulfillment of civic duties, and yes, civil disobedience). Both “negative” and “positive” are part of messy human life and important experiences for kids and teens. Isolating students from “negative” experiences would be unhelpful, setting them up for disillusionment and lack of resilience later when they find that “negative” experiences are common in life.

Beyond that, if kids are inclined to experiment with dishonesty (for example), then I’d rather they do so now, as kids, rather than as adults, when the consequences may be more severe.^38^

Yes, I think our school functions “relatively well” as human communities go. Even so, the school’s current project of collective self-examination and transformation illustrates that “relatively well” leaves plenty of space for continual improvement (Jim, 2024-08-17, Feedback).

Another example of existential civil disobedience involves the intentional breaking of boundary rules of wandering to a nearby creek or going to woods beyond yellow or red ropes (see the school rules in the Appendix). Although the rule transgressing students understand this safety rule, they have an existential need for contemplation alone or with friends in nature without staff members.^39^ Also, asking staff members is impractical because they are often busy. “I feel like multiple times it’s been a problem when I just tend to like exploring a fair bit. Like when I first came to the school, I was like, ‘Oh wow, I’m gonna get to spend the whole day in the woods, just exploring, going down to the creek.’ [But] with the creek, you can’t go down there without a staff member present^40^” (Linda, student, Interview).

A junior staff member reported a school ban on running inside the school building^41^ as a “bad” school rule, especially for very young children, who, according to this staff member, have a biological need to run often, “I disagree with that rule. I think that that doesn’t come very naturally to little kids. I think that they have boundless energy, and it’s almost natural for them to run, skip, and hop. And as we get older, we get more sedentary, and that just doesn’t appeal to us [anymore]. So, I’m not a fan of that rule. As a staff person, I feel that way. And as a parent, I feel that way. I think that was probably a rule back when I was a student, too” (Brook, junior staff and alumnus, Interview). Several interviewees reported that little children constantly violate this rule to the point that others rarely write them up to the JC. It is unclear how much little children’s disobedience of this school rule is principled. But, after discussing the Little Bryan case above, I do not automatically dismiss this possibility. If this were the case, I would qualify it as existential civil disobedience.

As I mentioned above, I am not reporting other cases of existential civil disobedience to avoid getting some students in trouble. I must conclude that existential civil disobedience, with its strategy of secretly principled violation or sabotage of a “bad” school rule, is the most prominent type of civil disobedience at this democratic school (cf. Corsaro, 1990 – the study about the underlife of the nursery school).

Through the interviews, I did not find any explicit cases of safeguarding the civil disobedience approach when civil disobedience is the last reserve against authorities’ grievous abuses, probably because, fortunately, such grievous abuses have not been encountered at the school.

However, I found some cases of expedient civil disobedience. Thus, a senior staff member (Jim Rietmulder) reported consciously violating a Circle School rule banning running indoors because of an emergency of “the sighting of a stranger walking on the far side of the campus near the swingset where some young children were playing (on our current campus). I was called to JC [the Judicial Committee] the next day, having been written up by a student for running indoors. I don’t remember who wrote me up, but I do remember that I and the JC understood that the complaint writer took some pleasure in writing up a staff member. After hearing my explanation, the JC discussed it and closed the case without charging me with any rule violation” (Jim Rietmulder, senior staff, Interview). It is interesting that Jim mentioned “some pleasure in writing up a staff member,” which may reflect somewhat uneasy relationships^42^ between the school staff and students, which I concurred with the interviews with the students, but these findings about school democratic governance were beyond my current investigation of civil disobedience.

Abigail (novice TCS student) commented in her feedback on this paragraph above: “In many ways, it feels like a rebellion. I would almost classify writing up a staff as its own type of civil disobedience.” Yes, I agree with Abigail that it is a form of civil disobedience because it hijacks a school practice to disrupt the life of the staff. I qualify this type of civil disobedience as instrumental because its tacit goal seems to be sharing the students’ pain because of the staff’s insensitivity to their needs. It may also suggest a lack of an open forum and legitimate channel to share students’ grievances with the staff. However, several staff members (Samantha, Jim, and Cody) commented that Abigail is a relatively new student at TCS, coming from conventional schools, and she might not fully understand how TCS works. Novice students have diverse trajectories of getting settled at and fully understanding a democratic school like TCS. In their views, Abigail’s overcritical views may reflect her trajectory and struggles.

A student (Nathan, Interview) also reported a case of expedient civil disobedience. The school property includes woods. According to a school safety rule (see in Appendix), the territory where students can freely walk is marked by yellow ropes.^43^

There’re yellow ropes that like mark the school boundaries. And you can’t go across the ropes like at all. So like, if I were to lose something, there’s been like numerous times where one of my things has rolled out of bounds or something, and I can’t go get it. It’s on bounds. I have to go like ask a staff, I go get my things. ... Like if you lose a personal item out of bounds by mistake or something, you can just go get it.

If a student needs to cross the yellow rope, they must be accompanied by a staff member or another student, “within earshot, or they have a cellphone with the school’s phone number saved in it. There is a red rope much deeper in the woods [10 feet from the creek] that requires staff presence to cross” (Cody, junior staff and alumnus, feedback). However, sometimes, students intentionally violate this rule because of expediency (Nathan, Interview). The student referred to this safety rule as “bad,” although they did not try to change it because, according to the student’s observation, students’ changing school rules was often unsuccessful and costly at the school.

As to the students’ normative beliefs, neither extreme civil disobedience nor extreme civil obedience was accepted by the interviewed students. Extreme civil disobedience is a belief that people must be governed exclusively by the laws they consent to (libertarianism). Extreme civil obedience is a belief that in perfect democratic governance, civil disobedience is not needed (democratism). Neither of the seven interviewed teenage students liked having only those school rules that everyone in school agrees with (i.e., the libertarian extreme of civil disobedience). One student explained that when all people agree with the rules, the rules are not needed. At the same time, neither believed that it was possible to create such a democratic system that would generate school rules that could satisfy everyone (i.e., the democratic extreme of civil obedience). Thus, they seemed to admit that a legitimate imposition of rules, norms, solutions, and values is necessary on a disagreeable minority via democratic governance.

The interviewed teenage students did not agree that even in a perfectly designed democratic governance system or practice, it is possible to change a disliked rule. In my interview with Alan (novice TCS student), he listed the four following reasons in the interview: 1) there may not be enough people to support the change (cf. “the tyranny of the majority”), 2) the person may not be skillful enough to articulate the reasons for the change or propose a good alternative rule, 3) the disliked rule might be super necessary for the school’s survival or required by the authorities outside of the school (e.g., legal issues), and 4) it may take prohibitively long take to make the change, so the person gives up on it.

It is interesting that when I offered the interviewed teen students a hypothetical example of them sitting at the school’s Judicial Committee and judging a proven transgression of a “bad” school rule – “bad” also from their own point of view – by another student at the school, they either considered to dismiss the brought case or, at least, to give minimum or no punishment (just warning). However, when asked if they disagreed with the accused student that the transgressed rule was “bad,” then they would charge the accused with the transgression and consider giving the accused a harsher sentence for an intentional violation.^44^ They would also advise the rule transgressor to try to change the rule, which the transgressor viewed as “bad.” The latter was surprising to me because all the students complained about the current democratic governance in the school. Pam (student) commented on my last sentence in her feedback: “This is a good point. I think the reason students still recommend going to school meetings is because we feel we have made a commitment to the school community, which requires us to use the tools provided, however flawed, to enact change. To me, this indicates that we haven’t completely given up on democracy at school.” Abigail (novice student) commented on this entire paragraph in her feedback: “I think we don’t know any other way to change a rule. Any civil disobedience is often unsuccessful and has bad consequences.” The reluctance of interviewed teens to acknowledge their peers’ civil disobedience when they disagree with it highlights their unwillingness to grant autonomy to their peers. Autonomy is a person’s freedom recognized by others, even when there are disagreements about actions within that freedom. It looks like that the interviewed TCS teens do not recognize their peers’ autonomy for civil disobedience by acknowledging only those peer civil disobedience, which they agree with.

#### Does the Democratic School Teach Civil Disobedience?

The first answer that comes to my mind is no, at least not directly. There is no public discourse of civil disobedience at the school, which was understood initially by all my interviewees instrumentally as a non-violent public protest way of changing society. However, my sharing of diverse approaches and understandings of the concept of civil disobedience, especially the existential approach of trying to live according to one’s own conscience and way of life, resonated with all of the students and junior staff members I interviewed. Existential civil disobedience is attractive not only to them but also to some of them who have been practicing it in and out of school.

Indirectly, however, there is some evidence that students may learn from each other the existential civil disobedience. Two junior staff members who were students at the school at the same time 17 years ago remember in their interview with me:

Cody: … I grew up a rule follower and have since come to be more comfortable with choosing to color outside of the lines a little bit when I wish to. Sometimes that’s rules, but sometimes that’s just expectations. … It’s not all Circle School, but there’s a part of me that wonders that knowing when to color outside of the lines maybe was, um, nurtured by The Circle School.

Samantha: I have a distinct memory of you as a teenager. Coming to church with me once, 'cause my parents are pastors. There’s a point where everyone goes up to get communion. And I turned to Cody and he’s like, “I’m not going up there!” And for me, it was like, “Whoa, you can just not do that! You can just not go.” I remember just being like [thinking], “Wow, you can just not do the norm thing, even though everybody else was going.” Growing up in that environment, it flew [in] the opposite direction that everyone else was going. You physically stand out when you don’t participate. And yet there was something there for you where it’s like, “This isn’t for me, and I’m not gonna do it.” It wasn’t for me either, but I just always did it. [For me,] that was a switch at some point, maybe in culmination with the school where it’s like, “Oh, you don’t have to do whatever it is that you don’t want to do.”

Cody: That connects back to doing what you need to do to be right with yourself.

Samantha: I think so.

Cody: That’s interesting. I don’t remember [that].

Samantha: I do. I remember it very well.

Cody: Well, that reminds me of when I was in public school [before] I started attending The Circle School in eighth grade. For many years leading up to that, we would say the Pledge of Allegiance each morning. I would never say “under God” – I was silent for that part. Yeah. I just didn’t wanna say it. I didn’t want attention for it. I’m sure I didn’t want attention for it or to change anything. I just didn’t; it was what I needed to do… to be right with myself.

Like Cody, another school alumnus (but not a school staff member), also noticed a change in herself regarding her blind following the authority rules, but she was not sure if this could be attributed to the influence of the school or her becoming an adult.

Brook, a junior staff and a school alumnus, also agreed that the school nurtures the existential type of civil disobedience to stand up for one’s right to be oneself. When I asked her how the school nurtures it, she replied:

I think that what I did develop at The Circle School was a sense of respecting and trusting my intrinsic voice. Like, you know, making decisions regardless of what other people thought or roles that I felt like were necessary or right for me, you know? Or even in going into adulthood, like [for example], dropping out of a PhD program because I felt like I was doing other richer and [more] interesting things in my life at that time, and I didn’t wanna finish my PhD. I felt like this inner empowerment to make those decisions for myself, even though it wasn’t traditional, or [what was expected of me, or in the face of disapproval or social pressure].

I know that my time at The Circle School as a student, back when I was 9 years old, 10, 11, 12, all those things really strengthened that part of me and that, that resiliency and that sense of empowered to make those decisions regardless of what others think or what the roles are, what society standards are, that kind of thing. But I felt like I was always guided towards, or drawn towards, what felt right, you know, the right thing to do (Brook, junior staff and alumnus, Interview).

Studying Henry David Thoreau’s tractate on civil disobedience, Frédéric Gros commented that disobedience is the affirmation of one’s uniqueness, one’s own unique voice and place, something that Brook (Junior Staff) seems to talk about in the above quote: “Disobedience must arise from that point at which a person discovers themselves to be irreplaceable, in the precise sense of having this experience of the non-delegable, the experience that ‘this is for me to do' (*mea res agitur*), that I cannot devolve onto anyone else the task of having to think the true, decide the just, and disobey what to me appears insufferable” (Gros, 2020).

When I asked Brook (junior staff member and alumnus) what school experiences and practices promoted this development of her unique voice, sense of personal righteousness, and self-confidence, she pointed out: “The emphasis on self. Yeah. The emphasis on self. Like, you [are] knowing what’s best for you, knowing yourself more than anyone else. And that no one should be deciding what you should be doing with your life, or how you can live it the most joyfully, or what brings you the most happiness, or how best you can participate in this society or community. Only you know that about yourself” (Brook, junior staff and alumnus, Interview). Deciding and following what is good for me by a student recognized by others is exactly missing in conventional and progressive schools based on educational paternalism (Matusov, 2024c, d, 2025, in press). This again reverberates with Gros’s analysis of Thoreau: “no-one … can be me ‘in my place.’ … No one can think in my place, and no one can decide in my place what is just or unjust. Likewise, no one can disobey in my place.”

## Conclusion

What did I learn from my journey examining civil disobedience in general and civil disobedience in a democratic school specifically? The three open symposia with my American and Russian colleagues helped me define civil disobedience as principled disobedience. However, the disobedience principle does not need to be verbalized; it can be articulated through a young child’s emotions, gestures, and actions, as the story of Little Bryan shows. During the Open Symposia, I learned that some democratic educators might think that civil disobedience is not needed for a well-working democratic school governance. Another important finding emerging from these Open Symposia was that neither democratic educator could come up with an example of civil disobedience in democratic schools. Finally, disobedience caused by a cultural misunderstanding is not a case of civil disobedience.

From my examination of the literature, I learned that there are at least four types of civil disobedience, while before, like many people, I could only imagine the following one: using civil disobedience as a political tool for making society better. This has been a hegemonic understanding among all participants of my research in a democratic school, which is probably influenced by the civil rights and anticolonial movements of the twentieth century. Although a case of instrumental civil disobedience – a female teenage student’s public protest of societal sexism in 2007 – was reported by school members, it seemed to be extraordinary for its participants, who were aware of the protest, and it was unsupported by the school despite its espoused commitment to “practicing life.” This use of civil disobedience as a public protest political tool disrupting the local sensibilities about the public female dress code was viewed as dangerous for the school to afford for its survival and/or too silly, hypocritical, and immature.^45^ The school, as represented by the voice of a senior school staff member, is very radical educationally, which he feels must be compensated by its cultural conservatism and conformism to the local and broader cultural values and sensibilities: “One can choose their own battles to fight.” It sounds like the school’s commitment to students’ practicing life is limited only to society’s and local-community’s cultural status quo. I think TCS staff’s concern about the school’s survival is not unreasonable (e.g., see the attempt of the UK government to close down the Summerhill democratic school in 1999, Vaughan et al., 2006). However, the exact boundary of this concern is debatable and should probably be the subject of ongoing debate at the school, which may or may not go to the school’s democratic governance involving the students.

The literature revealed to me the other three types of civil disobedience based on their goals and functions besides hegemonic instrumental civil disobedience as a tool for improving society. Existential civil disobedience prioritizes the person’s following their conscience and/or right way of living. Besides one’s conscience, it may involve dignity, ecology, sympathy, justice, deep urges, identity, well-being, privacy, and so on. Such “bad” school rules^46^ involve: keeping the lights on everywhere in the school building all school day (surveillance violating teen privacy, mistrust), preventing teens from wandering in the woods and to a creek (an urge for contemplation and solitude in nature), not running inside of the school building (violation of little children’s ecology), not allowing to sit on counters (violation of body ecology), and so on. This type of civil disobedience was the most commonly felt and reported by the students, although they did not initially recognize it as such. In contrast to Thoreau, who called for open existential civil disobedience in order “to live in truth with yourself” (Havel & Vladislav, 1989), the students at the school practice their civil disobedience privately by violating or sabotaging the disliked school rules secretly in order to avoid being written up to the Judicial Committee, which might lead to school punishment and a bad reputation at school (and, thus, to the limitations of their freedoms and desired activities based on the staff’s cooperation). The students sabotaged the school rules by dimming down the lights, sneaking to the creek or woods, secretly sitting and lying down on forbidden counters, and so on. For them, secretly living in truth with themselves was more important than public living in truth with yourself.

The expedient civil disobedience of using disobedience to preserve the spirit of the enterprise or a task at hand was practiced, described, and institutionalized in the Prussian military in the middle of the nineteenth century. I found a few cases of this type of civil disobedience in the school: the staff intentionally and principally violated the no-running school rule to chase a stranger who might jeopardize school safety, students occasionally crossed to the forbidden territory for them to quickly get objects outside the allowed boundaries, and students violated the whispering in the library rule when nobody who might object was around, and so on. All these infractions were not publicized but, when noticed, were considered to be excused or minor, not worth referring to JC. Through all my interviews with students, staff, and alumni, I got the impression that the school is increasingly trying to move away from the literalism that was more common in the past.

Finally, and fortunately, I could not find any cases of safeguarding civil disobedience aiming at preventing worse instances of abuse and mistreatment at the school. It may mean that either The Circle School (TCS) is a very safe place for its participants that does not require safeguarding civil disobedience, or it is such an unsafe place that even safeguarding civil disobedience might be super dangerous. I think it is the former. The Circle democratic school promotes a strong practice and sense of authorship and ownership of education and life (“practicing life” in terms of a school founder Jim Rietmulder, 2019) in its students, which, I think, is the primary mission of the school. “Practicing life” is practicing the students’ authorial agency. I hypothesize that this makes the students reflective, thoughtful, and critical. As one of my undergraduate teacher education students who met TCS children observed, and I concur with her observation, “I’m amazed how thoughtful the TCS kids are about themselves, their lives, and their education. They know what they want and why. They’re interested in life. Even little kids. I feel that they’re much more mature than we [i.e., undergraduate students, future teachers] are.” Through the limited and imperfect processes of democratic self-governance and the Judicial Committee (JC), TCS children can publicly raise grievances about what bothers them and by whom. 

Stronach and Piper (2008) studied a similar inquiry at Summerhill, another democratic school located in the UK, into the reasons for the absence of severe abuses in the school during its 85-year existence (at the time of their research). The researchers hypothesized that it was a result of a “benign panopticon" – total mutual surveillance of and by its participants, who can bring any digressions to the School Meeting for the public discussion, judgment, and sentencing (if necessary): “Summerhill is an almost perfect panopticon, incapable of secrets” (Stronach & Piper, 2008, p. 13). 

I respectfully disagree with this hypothesis because I do not believe that any mechanical system, especially based on total distrust and surveillance, can successfully prevent severe abuse – such a system is a severe abuse in itself. A “benign panopticon,” aiming at creating an internal policeman in people’s heads (Foucault, 1995), is a misnomer. Rather, I argue that a good record of many democratic schools being free from severe abuses is based on their successes in promoting their students’ authorial agency and building a civil society^47^ inside these democratic schools. Similarly, Jim Rietmulder (2019) insists that that the best protection that kids have in the world outside of school in general is their self-awareness and their confidence in their own autonomy. Another empirical study is needed to test these two alternative hypotheses explaining the absence of severe abuses in democratic schools.

This was reflected in the finding that the reported instances of existential civil disobedience were more numerous than instrumental and expedient civil disobedience in The Circle School. Instrumental and expedient civil disobedience is driven by mostly external forces rooted in society or activity. In contrast, existential civil disobedience is driven by internal, personal forces. The School affirms its students’ personal interests, personal lives, personal judgments, personal values, and personal voices—both private and public. In turn, it promotes a sense of human dignity in the students as unique human beings with unique aspirations, demands, worldviews, feelings, and values. Lev Tolstoy (1968) argued that self-recognition, self-affirmation, and self-legitimatization of human dignity are the basis of the existential^48^ civil disobedience, defined as a person’s insistence on living in truth with oneself, which must be recognized by others.

The students reported some problems with democratic governance school meetings, which was somewhat corroborated by junior staff who were students at the school in the past (not included in this research report). Although, in my judgment, the problems with the school’s democratic governance are serious and worth addressing by the school, I believe that solving these problems won’t eliminate the need for civil disobedience in the school, as all students suggested. The tyranny of the majority, when one student or a minority of students (and staff) cannot convince or impose their will on the school, will still prevail in any perfect or near-perfect collective self-governance project. The existential responsibility for one’s own life, which may go against the will or needs of the school, will remain. The expediency and urgency of the spirit of the enterprise or a task at hand, often in unique and unpredicted contexts, will run against well-thought-out rules. Hence, both civil obedience and civil disobedience are needed in well-designed school democratic governance. Public school forums on the merits and limitations of civil disobedience and disobedience can be helpful. One avenue for such a public forum at the school is the Judicial Committee (JC), where potential violations of the school rules are investigated. JC may ask the accused whether the breach was intentional and, if so, why; if the accused disliked the violated school rule and why; whether the accused considered changing the rule and, if not, why; and so on.

The absence of such public forums about civil disobedience at the school does not necessarily mean that the school does not promote civil disobedience as its “hidden,” or better to say unintended, curriculum. School alumni report that the school’s trust in the students’ authorial judgments about what is good for them nurtures the students’ ability to confidently stand by what they feel is right, which includes existential civil disobedience. This finding makes me hypothesize that excessive paternalism and, especially, educational paternalism, when parents, teachers, politicians, experts, and scientists claim that they know better what and why needs to be studied than the students themselves, suppresses the students’ authorial agency and, thus, inclination and ability for civil disobedience. Democratic education, based on the student’s right to decide whether to study, what to study, how to study, why to study, with whom, when, and so on, opposes educational paternalism and seems to promote the authorial agency in the students, positioning the students as authors for their own education and life. Civil disobedience is a byrpoductive curriculum at The Circle School. Frankly, this is another research finding that I did not anticipate because I expected that public dialogues on civil disobedience were necessary for this development.^49^

I believe that the tension between civil obedience to democratic governance and civil disobedience is inherent in human life and cannot be solved in any universal way, either by law, procedure, principle, philosophical maxim, technical formula, or via some perfect organizational structure as, for example, democratic governance alone. It requires an authorial judgment, a brave deed, taking personal responsibility from a person, and free and open democratic dialogic deliberation from society. A solution to such tension is often contextual, problematic, and controversial. Democratic schools seem to prepare their students to consider this tension well by nurturing the students’ authorship and ownership of their education and life. However, I did not find any evidence for a free and open democratic dialogic deliberation of this tension from the school’s democratically governed society – not in terms of solving this tension between civil obedience to democratically accepted school rules and civil disobedience to them once and for all but in terms of the awareness of the unavoidability of such tension in democratic self-governance.

Finally, in his feedback on a previous draft of my paper, Cody (junior staff and alumnus) provided a keen list of limitations of my research – I greatly appreciate his critical contribution to my research:

I think your own synthesis of the various perspectives and themes is required in order to find deeper meaning and avoid publicizing one-sided retellings of the school’s internal affairs. Such synthesis would require contextualizing student dissatisfaction with "bad rules" in a fuller context that includes various intersecting forces:

- **Teenage** desire for greatly increased independence, plus righteous, passionate rejection of restrictions on independence;
- **Societal** rejection of autonomy and independence for youth, heightening in our current cultural climate;
- **Staff** endowed with the responsibility to preserve the space where students receive much greater autonomy, independence, and authorial agency in their lives than they otherwise would — balancing student autonomy with societal norms and limits;
- **School Meetings** where these various ***strong*** forces intersect — and agreement over where the boundaries are is in a new round of evolution.

I agree with Cody’s critical points, which require more detailed future investigations.

I want to add to the list of research limitations that I would like to hear the voices of the students outside of the snowball self-selection of my research participants, especially the voices of the young students about their possible experiences and thoughts involving civil disobedience. Unfortunately, I could not find a way to enroll them in my research.

## Appendix: Excerpts From the School Lawbook

### 2411 Boundaries & Restricted Areas

#### 2411.02-SM the Boundary Rule

You must stay within the boundaries of the unrestricted school grounds. Yellow ropes, yellow stakes, and other markers are reminders of the boundaries, but you must stay within the boundaries even if the markers are missing. You may go out of bounds briefly to retrieve a lost ball or other objects that have inadvertently strayed, except that you may not enter or go over the creek, and you must follow rules regarding restricted areas.

Generally, the outdoor boundaries include:

- mowed yard
- northwest play field, but not the west-facing bank
- big hill
- blacktop play area (basketball area)
- ravine woods down to no closer than ten feet west of the creek, or the boundary marker, whichever is farther from the creek, and no farther south than the south property line that passes near the dumpster enclosure

For a map of the boundaries see the Appendix.

### 2447 Darkness

#### 2447.33-SM Lights Must Stay On; Exceptions

All room lights must be on at all times, except if the Facilities Manager has approved the lights being off for a specific activity, or if all of the following are true:

1. The lights are off for an “organized activity” (see below) and
2. The name of the individual or group turning the lights off, the time period for which the lights may be off, the date, and the reason the lights are off are all posted on a sign on the door of the affected room, and
3. The lights are turned back on at the end of the activity and the sign is removed.

For the purpose of this rule only, an “organized activity,” is one for which any one of the following is true:

- A corporation or committee (includes an individual member) is turning the lights off for an activity which is in line with the corporation by-laws or the committee’s charter, or
- An individual or group has reserved the room for purposes which require the lights off, or
- An individual or group has reserved the room for purposes which are in direct accord with a need to turn the lights off, even if the need was unforeseen, or
- The organized activity is under the direction of any Clerk, performing activities related to the performance of the Clerk’s duties.

### 2455 Quiet & Silent Areas

#### 2455.36-SM Library: Silent

During the school day you may use the library only for silent, still activities, except for occasional, brief whispered interactions. Library Committee business is exempt from this rule. The Aesthetics Committee may make other occasional exceptions to this rule. You may not hide or seek in the Library during games of hide-and-seek or otherwise.

## Data Availability

No datasets were generated or analysed during the current study.

## Footnotes

1 At the end of 2024, there are at least 367 democratic schools around the world (44 countries), with about 32% of them located in the USA, https://www.educationrevolution.org/store/findaschool/democraticschools/ (however, I know democratic schools outside of this list, e.g., “The Green School” in Romania, – thus, this list is incomplete).

2 However, this option was used in the George Junior Republic – a democratically run youth enterprise (George, 1910; Light, 2020).

3 This was not a sexist statement by Lev Tolstoy. The Russian word “chelovek,” “чeлoвeк,” translated here in English as “a man,” is a grammatically male noun that requires a grammatically male pronoun. It embraces both men (“мyжчины”) and women (“жeнщины”).

4 See also (Solzhenitsyn, 1974) for a more modern articulation of this conviction.

5 The essay’s title refers to the Russian Tsar Nicholas I (1796–1855, reign 1825–1855), who was nicknamed “Palkin” for his cruelty (“palka” *пaлкa*, in Russian, means a “stick” – beating by a stick was a common punishment in the tsarist army in that time).

6 I could not find this quote in Levy’s book “If This is A Man” (Levi, 1959), to which this quote is widely attributed.

7 Summer Ecological School (LESh, 1990-present) is a non-profit summer school for students in grades 6–11 (ages 12–18). Takes place annually in July and August. The school is represented by five departments: biology, physics, mathematics, humanities, and medicine, https://ru.wikipedia.org/wiki/%D0%9B%D0%B5%D1%82%D0%BD%D1%8F%D1%8F_%D1%8D%D0%BA%D0%BE%D0%BB%D0%BE%D0%B3%D0%B8%D1%87%D0%B5%D1%81%D0%BA%D0%B0%D1%8F_%D1%88%D0%BA%D0%BE%D0%BB%D0%B0.

8 https://www.nytimes.com/2015/09/04/us/kim-davis-same-sex-marriage.html

9 https://www.nytimes.com/2024/01/04/us/kim-davis-marriage-licenses-legal-fees.html

10 Unfortunately, neither Melissa Bradford nor I could find this essay among the vast literature left by Danny Greenberg so far. We keep searching for it.

11 I found a place where Danny Greenberg explicitly argues *against* a consensus in Democratic Education (and his Sudbury Valley School) as its principle: “Indeed, the mark of a democracy is the absence of consensus. Democratic procedure implies that all the conflicting alternatives be given a full and equal hearing, and be respected and allowed to persist even when their proponents are in the minority. In a democracy, consensus is a rare and short-lived accident, as this country found in the 1960’s. Repeated consensus is always a symptom of powerful communal pressure to force the dissenting minority to abandon its position and accept the prevailing view” (Greenberg & Sadofsky, 2013, p. 141). If Melissa Branford remembers correctly, it may reflect either inconsistencies or changes in Greenberg’s thinking. Jim Rietmulder [feedback, 2024–07-05]: “I agree with Danny [Greenberg] that the absence of consensus is a hallmark of large-scale democracy, but democratic schools are small-scale and characterized by categorically different politics and social dynamics. School-scale democracies are thickly connected networks of interpersonal relationships. Nation-scale democracies are not. As a consultant for 20 years, I worked with hundreds of small groups of adults in business, industry, and nonprofits. I found that virtually every small-scale group sought a general agreement and often achieved it on significant matters of policy and resources. I see similar dynamics in democratic schools: people seeking agreement and often finding it, sometimes after sustained dialogue. In short, democratic schools and democratic nations are different animals. The comparison is not apt (even though civil disobedience is present and worthy of research in both settings).”.

12 Despite their visible opposition, one can argue that Thoreau’s approach prioritizing civil disobedience over collectivity and “Greenberg” (in Bradford’s remembrance) – Habermas’s approach prioritizing democratic governance over personal civil disobedience are not so different if one believes in the omnipotent power of rational persuasion and rational discourse as Habermas does (but not necessarily Thoreau). These two approaches are both universalist.

13 Gros (2020) cited a quote from Zinn’s 1970 speech: “The problem is not disobedience, it is obedience” (see also Zinn, 2009).

14 https://www.vice.com/en/article/teachers-primary-secondary-school-protests-youth-strike-climate/

15 Also, it is important in more non-verbal cultures like, for example, Japanese or Myan (Rogoff, 2003).

16 https://arabaevksu.edu.kg/en/news/rossiyalyk-filosof-metodolog-petr-schedrovickiy-zhozhdordun-rektorloruna-lekciya-t-d

17 Jim Rietmulder [feedback, 2024–07-05]: “’I felt’ is the crux of the difference in my definition of civil disobedience: Little Bryan’s emotional response was at the root of his disobedience. But emotion is not principle, and I don’t think conceptualization happens “through … emotions.” I suppose civil disobedience entails both principle and passion in sufficient measure to motivate action. In spite of what I’ve said, it seems fine to expand the definition of ‘civil disobedience’ to include ‘emotional disobedience’ for the purposes of this study.”.

18 Jim Rietmulder [feedback, 2024–07-05]: “Yes, you’ve persuaded me of this. Change-the-world isn’t essential, but principle is.”.

19 Jim Rietmulder [feedback, 2024–07-05]: “I suppose this is true in a ‘pure’ or absolute democracy. In democratic nations and at The Circle School, participants are expected to generally submit to the rule of law, but not to the rule of the majority. In the U.S., for example, there is the Constitution and its Bill of Rights, which protect minorities from majorities. This point may or may not be relevant to your analysis of civil disobedience, but it’s essential in real-world ‘democracies’ such as The Circle School and the United States. (A leading edge of change for The Circle School is differentiating matters that are properly subject to popular vote from matters that are not.). Sovereignty vs non-sovereignty is an essential difference between nation-democracies and school-democracies. A nation is sovereign – self-governing – but a school is not. We call the school ‘self-governing,’ but it is only self-governing to the degree allowed by the sovereign nation within which it operates. With this in mind, the ‘participants’ voluntary subordination’ is to the school’s rule of law, and, in addition, the school’s “voluntary subordination” is to authorities beyond the school. I believe the school’s subordination or deference to external authorities accounts for most of the ‘invisible walls’ referred to elsewhere in this paper [actually, in an extended research report about findings that go beyond my initial inquiry of civil disobedience in a democratic school – Eugene Matusov]. Hmmm… I wonder if ‘invisible walls’ is the best conception of constraints that become progressively more evident as a person’s capacities expand. For example, as a tax-exempt nonprofit, the school is not allowed to lobby for legislation in the state legislature. This constraint is generally “invisible” and irrelevant to the school’s youngest children. Knowing and understanding the rules for the playroom is far more important and relevant to them. But, a teenager who has developed ideals and political opinions might very well need to know that the school must not lobby our legislators. Thus, the scope of a student’s abilities and interests may often determine what “walls” they will crash into and thus learn about. In a more expansive perspective, human development itself is essentially a spiral process of bumping into obstacles, gaining insight, expanding capacity, and then starting over with new obstacles that couldn’t previously have been reached.”.

20 This school democratic governance can be limited, making only certain decisions about the school life, or unlimited, making all decisions about the school life. The tension between limited and unlimited school democratic governance was portrayed and discussed by the adults and children in the documentary “Approaching the Elephant” (Wilder, 2014).

21 https://www.circleschool.org/

22 The educational term "hidden curriculum" refers to the “lessons” that are learned by the students but not explicitly intended by the formal education practice or system. These include the norms, values, and beliefs that are conveyed in the social environment of a school. The hidden curriculum can encompass various aspects, such as the reinforcement of social (in)equalities, gender roles, cultural norms, and the development of attitudes and behaviors that are not part of the formal curriculum or explicitly declared school practices (Giroux & Purpel, 1983; Jackson, 1968; Margolis, 2001).

23 In addition, Brook’s children have been attending the school, so Brook has spent time there as a parent for 6 years. She also spent 1 year as a staff volunteer, and then she was hired as a school staff member for 4 years in the 1990s. She was hired back as a staff member in 2022. Thus, her total staff experience is 7 years. In his feedback on my research report, a part about the school’s democratic governance that was not included in this paper, Jim Rietmulder raised a very good question about whether Brook should be considered as a “junior” or “senior” staff member. Based on Brook’s school experiences in different roles, she can be considered as “senior.” However, based on the school’s stewardship and ownership as school staff, I consider her a “junior” staff member. I wonder if Brook agrees with my characterization and justification of her current role in school. She has chosen not to reply.

24 Both senior staff members also had their children attend the school.

25 I placed the word “bad” in quotation marks with regard to school rules and norms to indicate that this judgment is not mine but by some of the research participants.

26 Jim Rietmulder [feedback, 2024–08-17]: “I don’t doubt the sincerity of this expression. Even so, I think the episode was profoundly educational in the best sense – and a good example of “practicing life” and the arduous work of finding agreement about how to live in the community.”.

27 Jim Rietmulder [feedback, 2024–07-05]: “This seems insightful to me. I wonder, too. And I wonder how much of their complaining is rooted in their sudden (this year) empowerment and discovery of the limits of that power – invisible walls suddenly made visible by liberation to go beyond previous boundaries (in their conventional schooling). You have to knock down the fence before you can see the fence behind it.”.

28 Jim Rietmulder [feedback, 2024–07–05]: “Hey! This reminds me (don’t know why) of Mickey’s Cookie on page 118 of *When Kids Rule the School* (Rietmulder, 2019, pp. 118–120) – a real case of civil disobedience.”.

29 I intentionally do not reveal these identifiable incidences of students’ rule breaking here to avoid putting the involved students in trouble with the school rules and staff, which may lead to worsening their stand in school and reputation. Abigail commented in her feedback on an early draft of the manuscript: “I noticed that you didn’t include direct rule breaking, I understand your concerns but if feels very important to the paper. I wonder if there is a way to include those instances safely.” I agree with Abigail about the importance of these rule-breakings for this research, but, unfortunately, I could not find a safe way of reporting them since it might affect the school reputation of these students and, thus, their possible future rights and privileges, which can be based on their school reputations.

30 Jim Rietmulder [feedback, 2024–07-05]: “An invisible wall made visible by the student’s growth and the action she took in response.”.

31 Jim Rietmulder [feedback, 2024–07-05]: “Yes, always balancing. Fortunately, for most students, most of the time, there’s no conflict between the two.”.

32 Jim Rietmulder [feedback, 2024–07-05]: “’Skewed’ is a loaded word here, implying unfair or distorted. I agree that staff, more than students, attend to the school’s ‘safety and survival’ – partly because staff have a legal and moral duty to do so, and partly because a broader and longer-term perspective tends to come with maturity.”.

33 In her feedback, Pam (student) commented on my wondering: “That’s definitely how it feels.”. Jim Rietmulder [feedback, 2024–08-17]: “I think this WAS ‘practicing life.’ What was balanced was student freedom, not ‘practicing life,’ so it might be more precise here to say, ‘at the expense of students’ freedom.’ Balancing individual freedom and the well-being of the school IS ‘practicing life!’ Sometimes that balance constrains individual freedom and brings disappointment – and that’s part of life.”.

34 Jim Rietmulder [feedback, 2024–07-05]: “I look forward to this. In the early years, the school was more vulnerable than it is now. Students more strongly felt the need to protect the school, so the gap between student and staff perspectives was less. I think the school is less concentrated on survival now, so students are freer to push the boundaries and feel less need to protect the school. By the way, I wouldn’t expect a teenager from a conventional school background (such as Abigail) to take the school’s perspective to a significant degree while still a newcomer to the school. They’re still rebounding from an oppressive school regime in which school staff [members] exercise comprehensive unilateral authority. The impulse to rebel against that seems natural and healthy to me.”.

35 Jim Rietmulder [feedback, 2024–07-05]: “The King (in the story about the Prussian officer) would not have hesitated to appoint Pam as a Major, knowing that she would know when to disobey an order (or a rule). Some rules (here and in the world at large) are calibrated to the least capable or least responsible. Pam (who has been in democratic schools all her life) is an excellent judge of when to disobey and why.”.

36 Jim Rietmulder [feedback, 2024–07-05]: “There may be some misunderstanding. When the lights were ‘dim[med] all the way down,’ the lights were still ON dimly; it wasn’t dark and didn’t violate the rule. Still, those who dimmed the lights may have THOUGHT they were violating the rule. I suppose that’s still civil disobedience if you aren’t breaking a rule, but you think you are!”.

37 Jim Rietmulder [feedback, 2024–08-17]: “I find the characterization of ‘junior’ and ‘senior’ staff (throughout this paper) to be crucially misleading. The distinction implies significance with respect to the topic of civil disobedience, but the rationale or significance is never explained. Without an explanation, the reader is likely to imagine (falsely) that there is a homogenous ‘junior’ staff perspective on civil disobedience and a homogenous ‘senior’ staff perspective on civil disobedience, and may also (falsely) imply an us-versus-them division among staff. In common understanding, the terms ‘junior’ and ‘senior’ indicate a difference in age or duration of staff service – i.e., ‘junior’ staff are younger and/or have been employed for fewer years. Indeed, the terms as used in this paper do accurately identify staff based on duration of service. But I think the more meaningful distinction – perhaps what the author really intends to get at – is the distinction between staff who were once students of this school versus staff who have never been students in a democratic school. This distinction seems more likely to indicate differences in thoughts about civil disobedience – that is, the ‘past-student’ perspective is likely to be meaningfully different from the ‘never-a-student’ perspective. The three staff members identified in this paper as ‘junior’ had all been employed as staff for less than three years at the time of the study, so the term ‘junior’ is accurate in that sense. But the coincidental fact that they were all students at some time in the past is what’s relevant to the topic of this paper – not the duration of their employment. I think the reader’s understanding would be improved if the terms ‘junior’ and ‘senior’ were avoided altogether. (The school’s newest staff member, whose staff service began after this study was mostly completed, would fit the ‘junior’ category, but he was never a student here and his views on civil disobedience in the school do not coincide with those of the other ‘junior’ staff. Firsthand experience makes the difference.). A parallel principle applies to students, but this paper makes no distinction between students who are relative newcomers and students who are ‘lifers’ (students who have been in this or similar schools for most or all of their schooling). Teens who are new to democratic schooling, coming from conventional (adult-directed) school backgrounds, are almost certain to have views about civil disobedience in school that differ greatly from teens who have been in (self-directed) democratic schooling for years. The difference is evident in this paper. In short, I don’t find views about civil disobedience to be meaningfully categorized based on age or duration of employment at the school. But, I do find meaningful generalizations distinguishing between a student perspective and a staff perspective. The school benefits from having some staff who hold both perspectives from first-hand experience.”. Eugene Matusov, [response, 2024–08-19]: “I separated the staff on ‘junior’ and ‘senior’ for the following reasons:

1. The authorship and ownership of the school;
2. The length of staff experience and knowledge of the school’s history and changes in its practice and values;
3. In contrast to the senior staff, all junior staff members have a “double perspective” as having TCS student and staff experiences.

At the same time, I treated all research participants as unique persons with distinguished and unique perspectives.”.

38 Cf. Japanese educators’ approach (Tobin, Davidson, & Wu, 1989).

39 Jim Rietmulder [feedback, 2024–08-17]: “Yes, I think it’s healthy for teens to have plenty of space and opportunities for contemplation and time apart from adults. Our school is fortunate to have a campus with nearly three acres of woods and a creek. Most of it is accessible without a staff member, but yes, the rule prohibits going closer than 10 feet to the creek (the red rope) without a staff member.”.

40 Jim Rietmulder [feedback, 2024–08-17]: “It’s true that school rules prohibit getting closer than 10 feet to the creek unless a staff member is present.”.

41 In my observation, the new school building makes running inside a relatively safe endeavor for the runner. However, little children running into other people can occasionally create a nuisance.

42 Jim Rietmulder, senior staff, [feedback, 2024–07-05]: “Or it could have been an especially easy relationship that accommodates teasing. More likely than both, the write-up was motivated by an emphasis on rules and concrete thinking that often prevails in middles. BTW, if the relationship were ‘uneasy,’ the student could have registered the complaint without revealing their identity. They didn’t do that, indicating they weren’t afraid of my retribution.”.

43 Jim Rietmulder [2024–07-05]: “No, the rule doesn’t require a staff member unless they want to go in the creek or within 10 feet of it – the red tape. Omit the reference to ‘accompanied by staff’ and this sentence is roughly true.”.

44 In her feedback, Pam (student) commented, “I have been thinking about this a lot because it does seem so hypocritical. My current thought is that this actually fits into the moral view of existential disobedience because the individual uses their personal moral code (i.e., ‘lives according to one’s own conscience’) to decide the ‘right’ outcome rather than applying a cookie-cutter sense of right or wrong over every situation.”. Jim Rietmulder, [feedback, 2024–07-05]: “What they [i.e., TCS teenage students] might not acknowledge is a pattern that has been troubling to some staff: teens on JC will often treat young children more harshly than their peers – often letting their peers off with a warning or simply dismissing the case. It’s troubling but not surprising that peers are acutely sensitive to their position in teen culture.”.

45 Jim Rietmulder [feedback, 2024–07-05]: “No, it wasn’t the ‘use of civil disobedience’ that raised alarms. Civil disobedience was not the problem. The problem was a teenage girl going topless in school – a problem of public image and a threat to the school’s license to operate. It would have been a problem whether or not it was an act of civil disobedience.”.

46 In his feedback, Cody (junior staff and alumnus) commented: This misses the nuance of reality. The “bad” rules generally represent areas of outside pressure (from government regulation/enforcement to market viability). The tension between doing something radical in education and remaining viable results in these compromises. Simply representing them as “bad rules” in violation of the child is inaccurate and misses an opportunity to more deeply assess the many forces at play that create conditions for civil disobedience. In addition to viability and survivability, there is great value in practicing life in an environment whose norms are reasonably aligned with those of the surrounding community/society. The ability to practice life is diminished as an environment strays further from the norms and values of the surrounding community/society.

47 “Civil society refers to the arena of uncoerced collective action around shared interests, purposes and values. In theory, its institutional forms are distinct from those of the state, family and market, though in practice, the boundaries between state, civil society, family and market are often complex, blurred and negotiated. Civil society commonly embraces a diversity of spaces, actors and institutional forms, varying in their degree of formality, autonomy and power” (Centre for Civil Society, 2006, p. 0). Civil society is absent in conventional and, to a bit lesser degree, in progressive schools based on paternalistic education.

48 He did not use this term; instead, he talked about conscience-based civil disobedience. I think the term “existential” civil disobedience is broader and, thus, more exact.

49 Jim Rietmulder, senior staff, [feedback, 2024–07-05]: “An insight I’ve gained from your study is that the authorial agency we value leads to greater awareness of and willingness to confront ‘bad’ rules and ineffective systems and practices. Thus, the better we are at promoting authorial agency, the more we will have to continue to improve the school.”.
